# Machine learning meets mechanistic modelling for accurate prediction of experimental activation energies†

**DOI:** 10.1039/d0sc04896h

**Published:** 2020-11-05

**Authors:** Kjell Jorner, Tore Brinck, Per-Ola Norrby, David Buttar

**Affiliations:** Early Chemical Development, Pharmaceutical Sciences, R&D, AstraZeneca Macclesfield UK david.buttar@astrazeneca.com; Applied Physical Chemistry, Department of Chemistry, CBH, KTH Royal Institute of Technology Stockholm Sweden; Data Science & Modelling, Pharmaceutical Sciences, R&D, AstraZeneca Gothenburg Sweden

## Abstract

Accurate prediction of chemical reactions in solution is challenging for current state-of-the-art approaches based on transition state modelling with density functional theory. Models based on machine learning have emerged as a promising alternative to address these problems, but these models currently lack the precision to give crucial information on the magnitude of barrier heights, influence of solvents and catalysts and extent of regio- and chemoselectivity. Here, we construct hybrid models which combine the traditional transition state modelling and machine learning to accurately predict reaction barriers. We train a Gaussian Process Regression model to reproduce high-quality experimental kinetic data for the nucleophilic aromatic substitution reaction and use it to predict barriers with a mean absolute error of 0.77 kcal mol^−1^ for an external test set. The model was further validated on regio- and chemoselectivity prediction on patent reaction data and achieved a competitive top-1 accuracy of 86%, despite not being trained explicitly for this task. Importantly, the model gives error bars for its predictions that can be used for risk assessment by the end user. Hybrid models emerge as the preferred alternative for accurate reaction prediction in the very common low-data situation where only 100–150 rate constants are available for a reaction class. With recent advances in deep learning for quickly predicting barriers and transition state geometries from density functional theory, we envision that hybrid models will soon become a standard alternative to complement current machine learning approaches based on ground-state physical organic descriptors or structural information such as molecular graphs or fingerprints.

## Abstract 2

Hybrid reactivity models, combining mechanistic calculations and machine learning with descriptors, are used to predict barriers for nucleophilic aromatic substitution.

## Graphical abstract

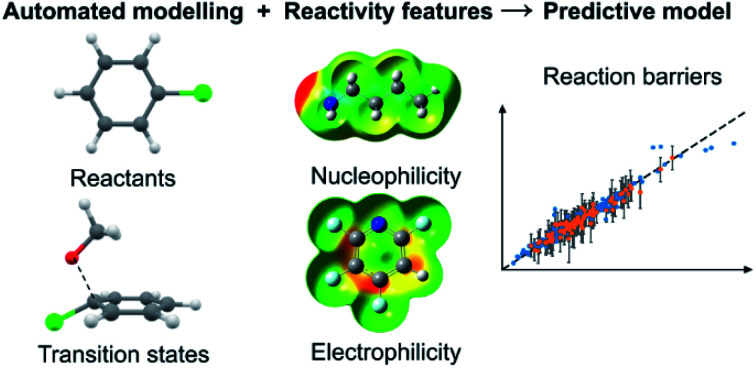

## Introduction

Accurate prediction of chemical reactions is an important goal both in academic and industrial research.^1–3^ Recently, machine learning approaches have had tremendous success in quantitative prediction of reaction yields based on data from high-throughput experimentation^4,5^ and enantioselectivities based on carefully selected universal training sets.^6^ At the same time, traditional quantitative structure–reactivity relationship (QSRR) methods based on linear regression have seen a renaissance with interpretable, holistic models that can generalize across reaction types.^7^ In parallel with these developments of quantitative prediction methods, deep learning models trained on reaction databases containing millions of patent and literature data have made quick qualitative yes/no feasibility prediction routine for almost any reaction type.^8^

**Fig. 1:**
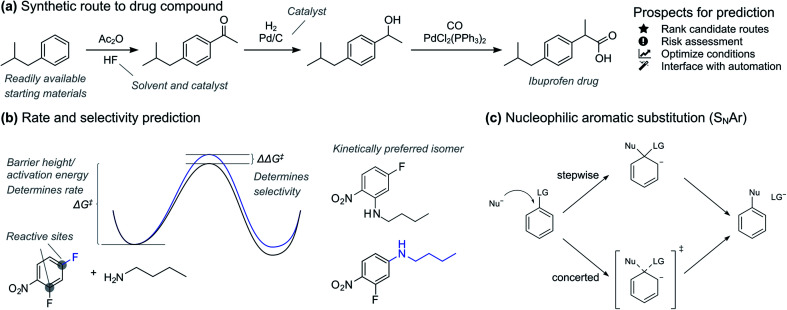
(a) Example of synthetic route to a drug compound. Prospects for AI-assisted route design. (b) Accurate prediction of reaction barriers gives both rate and selectivity. (c) The nucleophilic aromatic substitution (S_N_Ar) reaction.

In the pharmaceutical industry, prediction tools have great potential to accelerate synthesis of prospective drugs (Fig. 1a).^9^ Quick prediction is essential in the discovery phase, especially within the context of automation and rapid synthesis of a multitude of candidates for initial activity screening.^3,10,11^ In these circumstances, a simple yes/no as provided by classification models is usually sufficient. More accurate prediction is necessary in the later drug development process, where the synthesis route and formulation of one or a few promising drug candidates is optimized. Here, regression models that give the reaction activation energy can be used to predict both absolute reactivity and selectivity (Fig. 1b). Prediction of absolute reactivity can be used to assess feasibility under process-relevant conditions, while prediction of selectivity is key to reducing purification steps. Predictive tools therefore hold great promise for accelerating route and process development, ultimately delivering medicines to patients both faster and at lower costs.

**Fig. 2:**
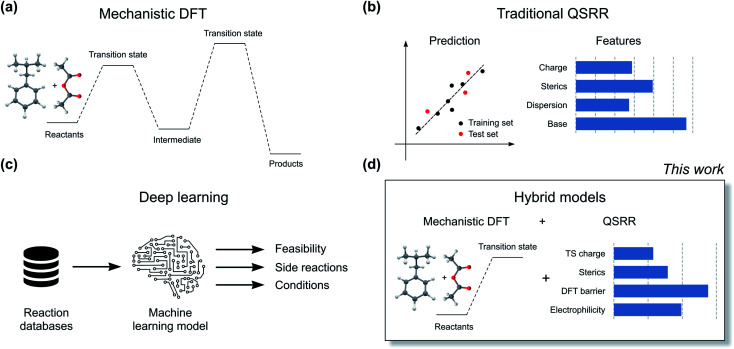
Different types of quantitative reaction prediction approaches. Mechanistic DFT (a) and QSRR (b) are the current gold standard methods. Deep learning models (c) are emerging as an alternative. Hybrid models (d) combine mechanistic DFT modelling with traditional QSRR.

The current workhorse for computational studies of organic reactions is density functional theory (DFT, Fig. 2a). Since rising to prominence in the early 90s, DFT has enjoyed extraordinary success in rationalizing reactivity and selectivity across the reaction spectrum by modelling the full reaction mechanism.^12^ The success of DFT can be traced in part due to a fortuitous cancellation of errors, which makes it particularly suited for properties such as enantioselectivity, which depends on the relative energies of two structurally very similar transition states (TSs). However, this cancellation of errors does not generally extend to the prediction of the absolute magnitude of reactions barriers (activation free energies, Δ*G*^‡^). In particular, DFT struggles with one very important class of reactions: ionic reactions in solution. Plata and Singleton even suggested that computed mechanisms of this type can be so flawed that they are “not even wrong”.^13^ Similarly, Maseras and co-workers only achieved agreement with experiment for the simple condensation of an imine and an aldehyde in water by introducing an *ad hoc* correction factor, even when using more accurate methods than DFT.^14^ These results point to the fact that the largest error in the DFT simulations is often due to the poor performance of the solvation model.

Machine learning represents a potential solution to the problems of DFT. Based on reaction data in different solvents, machine learning models could in principle learn to compensate for both the deficiencies in the DFT energies and the solvation model. Accurate QSRR machine learning models (Fig. 2b) for reaction rates or barriers have been constructed for, *e.g.*, cycloaddition,^15,16^ S_N_2 substitution,^17^ and E2 elimination.^18^ While these models are highly encouraging, they treat reactions that occur in a single mechanistic step and they are based on an amount of kinetic data (>500 samples) that is only available for very few reaction classes. Another promising line of research uses machine learning to predict DFT barrier heights and then use these barrier heights to predict experimental outcomes.^19^ A recent study from Hong and co-workers used the ratio of predicted DFT barriers to predict regioselectivity in radical C–H functionalization reactions.^20^ While these models can show good performance, the predicted barriers still suffer from the shortcomings of the underlying DFT method and solvation model. We therefore believe that for models to be broadly applicable in guiding experiments, they should be trained to reproduce experimental rather than computed barrier heights.

Based on the recent success of machine learning for modelling reaction barriers, we wondered if we could combine the traditional mechanistic modelling using DFT with machine learning in a hybrid method (Fig. 2d). Machine learning would here be used to correct for the deficiencies in the mechanistic modelling. Hybrid models could potentially reach useful chemical accuracy (error below 1 kcal mol^−1^)^21,22^ with fewer training data than QSRR models, be able to treat more complicated multi-step reactions, and naturally incorporate the effect of catalysts directly in the DFT calculations. Mechanistic models are also chemically understandable and the results can be presented to the chemist with both a view of the computed mechanism and a value for the associated barrier. As a prototype application for a hybrid model, we study the nucleophilic aromatic substitution (S_N_Ar) reaction (Fig. 1c), one of the most important reactions in chemistry in general and the pharmaceutical chemistry in particular. The S_N_Ar reaction comprises 9% of all reaction carried out in pharma,^23^ and features heavily in commercial routes to block-buster drugs.^24,25^ It has recently seen renewed academic interest concerning whether it occurs through a stepwise or a concerted mechanism.^26^ We show that hybrid models for the S_N_Ar reaction reach chemical accuracy with *ca.* 100–200 reactions in the training set, while traditional QSRR models based on quantum-chemical features seem to need at least 200 data points. Models based on purely structural information such as reaction fingerprints need data in the range of 350–400 samples. If these results hold also for other reaction classes, we envision a hierarchy of predictive models depending on how much data is available. Here, transfer learning might ultimately represent the best of both worlds. Models pre-trained on a very large number of DFT-calculated barriers^27^ can be retrained on a much smaller amount of high-quality experimental data to achieve chemical accuracy for a wide range of reaction classes.

## Results and discussion

First, we describe how the S_N_Ar reaction dataset was collected and analysed. We then describe the featurization of the reactions in terms of ground state and TS features. Machine learning models are then built and validated. Finally, we use the model for regio- and chemoselectivity prediction on patent reaction data, a task the model was not explicitly trained for.

### Reaction dataset

**Fig. 3:**
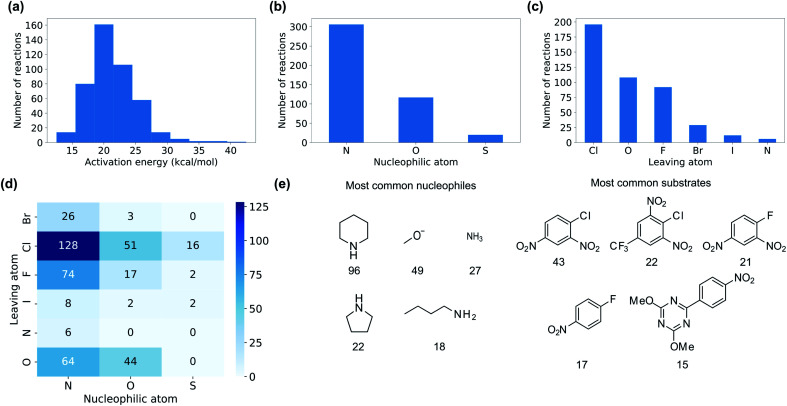
Distribution of (a) activation energies (b) nucleophilic atoms and (c) leaving atoms in the dataset. (d) Number of reactions in the training set for combinations of nucleophilic atom and leaving atom. (e) Frequency of the five most common nucleophiles and substrates in the training set.

**Fig. 4:**
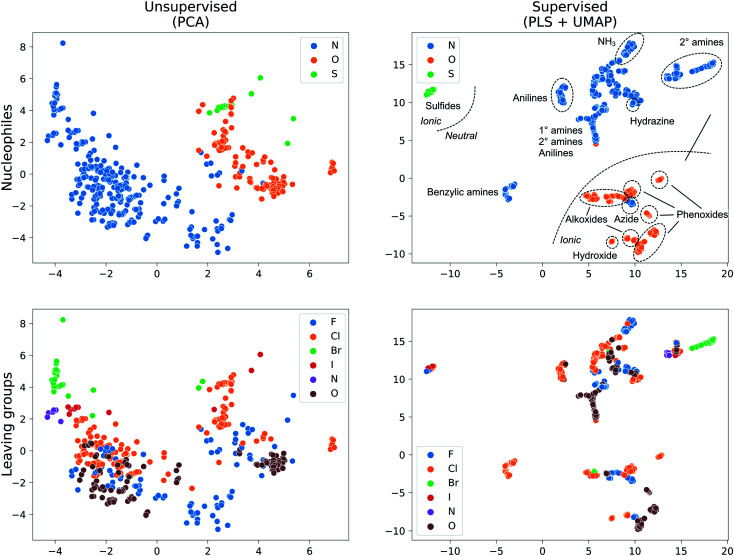
Dataset visualization with unsupervised (PCA) and supervised (PLS + UMAP) dimensionality reduction for the ***X***_full_ feature set.

We collected 449 rate constants for S_N_Ar reactions from the literature and ran 443 (98.7%) successfully through the modelling procedure. Of these 443 reactions, 336 corresponded to unique sets of reactants and products, of which 274 were performed under only one set of conditions (temperature and solvent) while 62 were performed under at least two sets of conditions. Activation energies were obtained from the rate constants *via* the Eyring equation at the reaction temperature, and were in the range 12.5–42.4 kcal mol^−1^ with a mean of 21.3 kcal mol^−1^ (Fig. 3a). The dataset is diverse, with nitrogen, oxygen and sulphur nucleophiles (Fig. 3b) and oxygen, halogen and nitrogen leaving groups (Fig. 3c), although the combinations of nucleophilic atom and leaving atoms is unevenly populated (Fig. 3d). The two most common nucleophiles are piperidine (96 entries) and methoxide (49 entries), while the most common substrates are dinitroarenes (Fig. 3e). A principal components analysis of the full feature space (*vide infra*) reveals a clear separation of reactions with respect to different nucleophile and leaving group atom types (Fig. 4). Supervised dimensionality reduction with partial least squares (PLS) to 5 dimensions, followed by unsupervised dimensionality reduction with the uniform manifold approximation and projection (UMAP) method^28^ yielded separated clusters with clear chemical interpretation (Fig. 4).

### Reaction feature generation

**Fig. 5:**
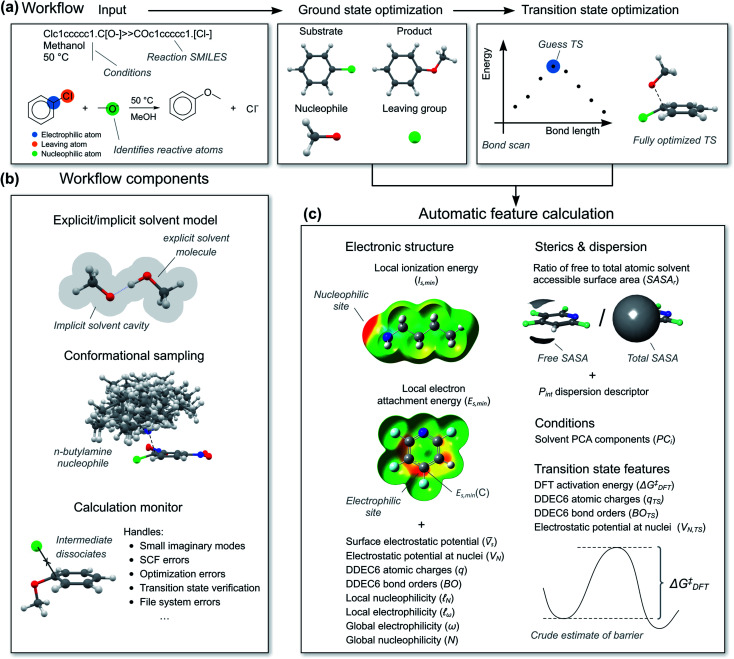
(a) Automatic workflow for calculation of reaction mechanism and features. (b) Important components of the workflow. (c) Features calculated for ground and transition states. Conformational sampling done with GFN2-xTB using the CREST tool, geometries refined with ωB97X-D/6-31+G(d) with SMD solvation. Final single points energies with ωB97X-D/6-311+G(d,p). Electronic structure features calculated with B3LYP/6-31+G(d).

To calculate the reaction features automatically, we constructed a workflow called predict-S_N_Ar. The workflow takes a reaction SMILES representation as input, deduces the reactive atoms and computes reactants, transition states and products with a combination of semi-empirical (SE) methods and DFT (Fig. 5a). Both concerted and stepwise mechanisms are treated and all structures are conformationally sampled, making use of the lowest-energy conformer (Fig. 5b). In the case of anionic nucleophiles, a mixed explicit/implicit solvation model was employed to reduce the errors of the computed barriers. The workflow also automatically calculates the quantum mechanical reaction features (Fig. 5c). When reactions corresponded to substitution on several electrophilic sites in a substrate, each reaction was treated with a separate calculation. Initially, we attempted to calculate the transition states with SE methods, but this was unsuccessful. Anionic nucleophiles are artificially destabilized by the lack of diffuse basis functions in the SE method, and the resulting potential energy surface is therefore highly distorted. We therefore used a more robust combination of SE and DFT as outlined in the ESI, Section 2.3.† There are still some limitations of our S_N_Ar workflow that could be improved in future work, such as treating counter-ions and acid and base catalysis.

For the hybrid model, we needed features for both the ground state molecules and the rate-determining transition state. We opted for physical organic chemistry features which would be chemically understandable and transferable to other reactions.^29^ We selected features associated with nucleophilicity, electrophilicity, sterics, dispersion and bonding as well as features describing the solvent. As “hard” descriptors of nucleophilicity and electrophilicity, we used the surface average of the electrostatic potential (*V̄*_s_) of the nucleophilic or electrophilic atom.^30,31^ (“Descriptor” and “feature” are here used as synonyms.) As “soft” descriptors we used the atomic surface minimum of the average local ionization energy (*I*_s,min_),^32^ as well as the local electron attachment energy (*E*_s,min_), which has been shown to correlate well with S_N_Ar reactivity.^33,34^ From conceptual DFT, we used the global electrophilicity descriptor ω and nucleophilicity descriptor N, as well as the corresponding local nucleophilicity and electrophilicity descriptors 
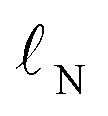
 and 
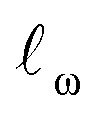
.^35^ These electronic features were complemented with atomic charges (*q*) from the DDEC6 scheme^36^ and the electrostatic potential at the nuclei (*V*_N_) of the reactive atoms.^37^ In terms of sterics and dispersion, we use the ratio of solvent accessible surface area (SASA_r_)^38^ of the reactive atoms and the universal quantitative dispersion descriptor *P*_int_.^39^ For bonding, we used the DDEC6 bond orders (BO) of the carbon-nucleophile and carbon-leaving group bonds.^40^ Solvents were described using the first five principal components (PC_1_–PC_5_) in the solvent database by Diorazio and co-workers.^41^ The most important TS feature was the DFT-calculated activation free energy (Δ*G*^‡^_DFT_), *i.e.*, a “crude estimation” of the experimental target.^42^ We also added *V*_N_, DDEC6 charges and bond orders at the TS geometry. We decided to not include the reaction temperature as one of the features, as reactions with higher barriers tend to be run at higher temperatures as they are otherwise too slow, and therefore correlate unduly with the target (see ESI, Section 5.6†). Atomic features are denoted with C (central), N (nucleophilic) or L (leaving) in parenthesis, *e.g.*, *q*(C) for the atomic charge of the central atom. Features at the TS geometry are indicated with a subscript “TS”, *e.g.*, *q*(C)_TS_.

We chose to investigate three main feature sets: (1) ***X***_full_, containing all the features (34 in total) for maximum predictive accuracy, (2) ***X***_noTS_ without any information from the TS, to assess whether hybrid models are indeed more accurate, and (3) ***X***_small_, which represents a minimal set of 12 features that can be interpreted more easily, with Δ*G*^‡^_DFT_ as the only TS feature (see ESI† for complete list). We also made two versions of ***X***_noTS_, excluding either surface electronic descriptors (***X***_trad_, missing *V̄*_s_, *I*_s,min_, *E*_s,min_ and *P*_int_) or traditional features (***X***_surf_, missing ω, N, ℓ_ω_, ℓ_N_, *V*_N_, *q*, and BO). As a comparison to the physical organic features, we investigated four feature sets that only make use of the 2D structural information of the molecule: the Condensed Graph of Reaction (CGR) with the *In Silico* Design and Data Analysis (ISIDA) descriptors^43^ (***X***_ISIDA,atom_ and ***X***_ISIDA,seq_), the Morgan reaction fingerprints^44^ as implemented in the RDKit (***X***_Morgan_),^45^ as well as the deep learning reaction fingerprints from Reymond and co-workers (***X***_BERT_).^46^ These structural features can be calculated almost instantaneously and are useful for fast prediction. We added solvent information to the structural features by concatenating PC_1_–PC_5_.

### Choosing the best machine learning model

We split the data randomly into 80% used for model selection (training set) and 20% used to validate the final model (test set). To compare the performance of a series of machine learning models on the training set, we used bootstrap bias-corrected cross-validation (BBC-CV) with 10 folds.^47^ BBC-CV is an economical alternative to nested cross-validation to avoid overfitting in the model selection process and also gives an estimate of the bias for choosing the model that performs best on the training set. We measure performance with the squared correlation coefficient (*R*^2^), the mean absolute error (MAE) and the root mean squared error (RMSE). We focus on the MAE as its scale is directly comparable to the prediction error. Error bars are given in terms of one standard error of the mean.

**Fig. 6:**
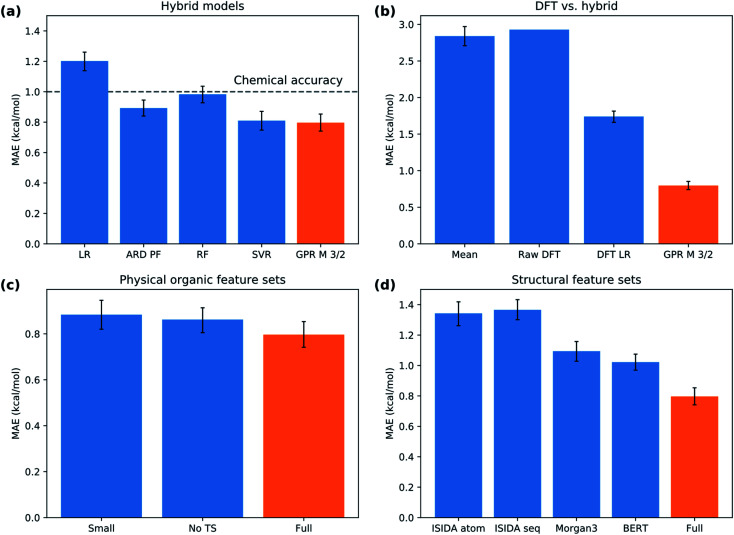
Model performance. (a) Selection of hybrid models of increasing complexity. (b) Performance of DFT *versus* hybrid model. (c) Comparison of physical organic feature sets. (d) Comparison of structural feature sets. Error bar corresponds to one standard error. Abbreviations explained in the text. The orange bar corresponds to the same model in all subplots, GPR_M3/2_ with the ***X***_full_ feature set. A comparison of all the models on the same scale is given in Fig. S6.†

**Fig. 7:**
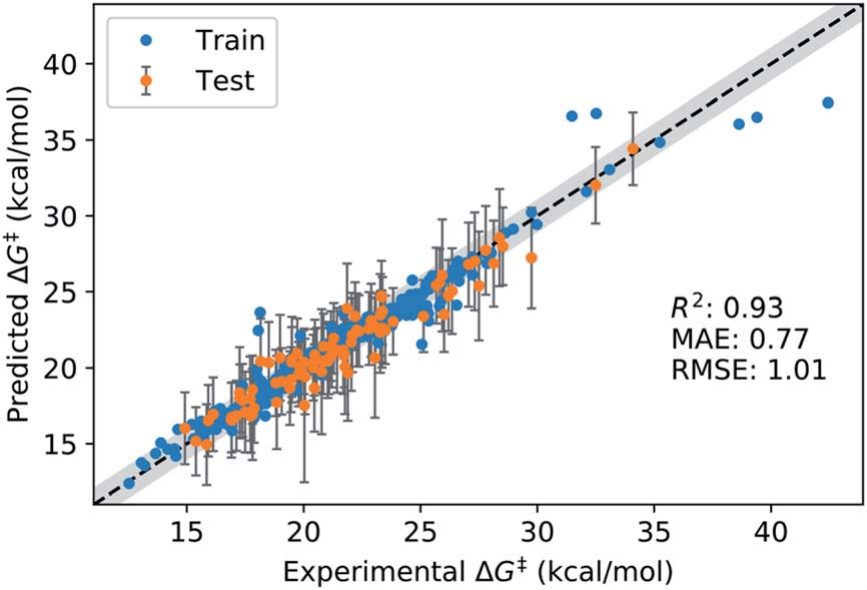
Performance of final GPR_M3/2_ model on the external test set. Coverage for test set: 99%. The grey band corresponds to ±1 kcal mol^−1^ with respect to the identity line.

The results of the model validation (Fig. 6a and Table S5†) show a clear progression from simpler models such as linear regression (LR) with a MAE of 1.20 kcal mol^−1^, to intermediate methods such as random forests (RF) at 0.98 kcal mol^−1^, to more advanced Support Vector Regression (SVR) and Gaussian Process Regression (GPR) models at 0.80 kcal mol^−1^. Most importantly, the best methods are well below chemical accuracy (1 kcal mol^−1^). In comparison, the raw DFT barriers Δ*G*^‡^_DFT_ show a high MAE of 2.93 kcal mol^−1^ and have the same predictive value as just guessing the mean of the training dataset (Fig. 6b). Compensating for systematic errors in the DFT energies by linear correction helps, but still has an unacceptable MAE of 1.74 kcal mol^−1^. Interestingly, simpler linear methods such as the Automatic Relevance Determination (ARD) can achieve the same performance as the non-linear RF when polynomial and interaction features of second order (PF2) are used to capture non-linear effects. The overall best method considering MAE is GPR with the Matern 3/2 kernel (GPR_M3/2_). This result is very gratifying as GPRs are resistant to overfitting, do hyperparameter tuning internally and produce error bars that can be used for risk assessment. We therefore selected GPR_M3/2_ as our final method and also used it to make comparisons between different feature sets. In the BBC-CV evaluation, it had an *R*^2^ of 0.87, an MAE of 0.80 kcal mol^−1^, and an RMSE of 1.41 kcal mol^−1^. Importantly, the BBC-CV method indicates a very low bias of only 0.02 kcal mol^−1^ for MAE in choosing GPR_M3/2_ as the best method, so overfitting in the model selection can be expected to be small. Indeed, GPR_M3/2_ shows excellent performance on the external test set, with a *R*^2^ of 0.93, a MAE of 0.77 kcal mol^−1^ and an RMSE of 1.01 kcal mol^−1^ (Fig. 7). The prediction intervals measuring the uncertainty of the individual prediction have a coverage of 99% for the test set, showing that the model can also accurately assess how reliable its predictions are.

### Performance of different feature sets

**Fig. 8:**
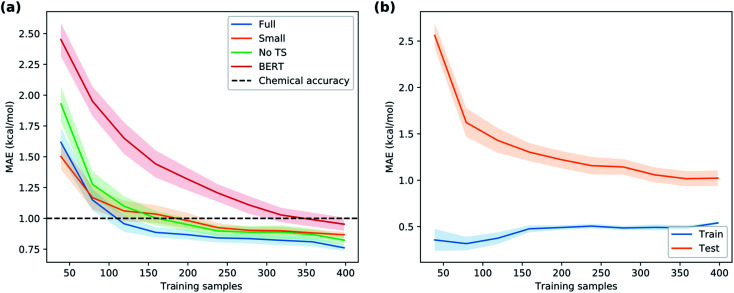
(a) Learning curve giving the mean absolute error as a function of number of reactions in the training set. (b) Learning curve to predict the DFT activation energies using the ground state features. Shaded regions correspond to one standard error.

Now, are hybrid models using TS features better than the traditional QSRR models based on just ground-state features? The validation results indicate that hybrid models built with the full feature set ***X***_full_ perform the best, but not significantly better than models built on ***X***_noTS_ without TS features (Fig. 6c). Also the model built on the small expert-chosen feature set ***X***_small_ shows similar performance. To investigate the matter more deeply, we calculated the learning curves of GPR_M3/2_ using the different features sets (Fig. 8a) on the full dataset. We see that all models indeed do perform similarly with the amount of training data used for the model selection (318 reactions), but that the hybrid models based on ***X***_full_ and ***X***_small_ seem to have an advantage below 150 samples. Indeed, the learning curve for predicting the Δ*G*^‡^_DFT_ based on ***X***_noTS_ also starts levelling off after *ca.* 150 samples (Fig. 8b). This indicates that the model is able to implicitly learn Δ*G*^‡^_DFT_ from the ground state features given sufficient data. It seems that there is still a residual advantage using the full hybrid model even with larger dataset sizes, although this advantage becomes smaller and smaller. For larger datasets it would therefore make sense to apply the “one-standard-error” rule and use the less complex model based on ***X***_noTS_ which is easier to implement. This rule states that a simpler model could be chosen in place of a better-scoring and more complex one if the score of the simpler model is within one standard error of the more complex model.^48,49^ But with fewer datapoints, it would instead make sense to use ***X***_full_ for maximum performance. Models built on ground state features lacking either surface features (***X***_trad_) or lacking more traditional electronic descriptors (***X***_surf_) showed worse performance (MAE: 1.00 and 1.10 kcal mol^−1^, respectively) than when both were included as in ***X***_noTS_ (MAE: 0.86 kcal mol^−1^). Therefore, both should be included for maximum performance and seem to capture different aspects of reactivity. It will be interesting to see if these trends with regard to dataset size hold up also for other reaction classes.

How good can the model get given even more data? Any machine learning model is limited by the intrinsic noise of the underlying training data, given in our case by the experimental error of the kinetic measurement. In the dataset, there are four reactions reported with the same solvent and temperature but in different labs or on different occasions. Differences between the activation energies are 0.1, 0.1, 0.5 and 1.6 kcal mol^−1^. The larger difference is probably an outlier, and we estimate the experimental error is on the order 0.1–0.5 kcal mol^−1^. In comparison, the interlaboratory error for a data set of S_N_2 reactions was estimated to *ca.* 0.7 kcal mol^−1^.^17^ It is thus reasonable to believe that the current model with a MAE of 0.77 kcal mol^−1^ on the external test set is getting close to the performance that can be achieved given the quality of the underlying data. Gathering more data is therefore not expected to significantly improve the accuracy of the average prediction, but may widen the applicability domain by covering a broader range of structures (*vide infra*) and reduce the number of outlier predictions.

### Models based on structural information

**Fig. 9:**
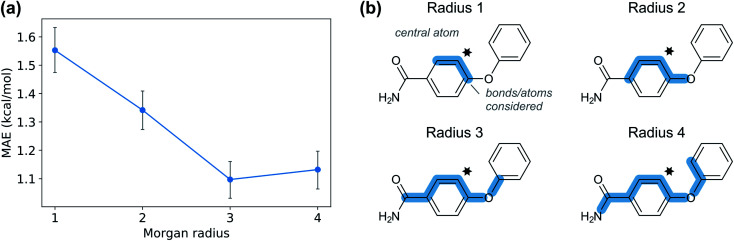
(a) Mean absolute error for GPR_M3/2_ as a function of Morgan fingerprint radius. (b) Atoms and bonds considered by Morgan fingerprints of different radius. A radius of radius three or more is needed to capture the effect from groups in the *para* position of the aromatic ring.

Given the time needed to develop both traditional and hybrid QSRR models, an attractive option is using features derived from just chemical connectivity. We investigated this option using the CGR/ISIDA approach with either atom-centred (***X***_ISIDA,atom_) or sequential (***X***_ISIDA,seq_) fragment features, as well as reaction difference fingerprints of the Morgan type with a radius of three atoms (***X***_Morgan3_). The results with GPR_M3/2_ show good performance using ***X***_Morgan3_, almost reaching chemical accuracy with a MAE of 1.09 kcal mol^−1^ (Fig. 6d). ISIDA sequence and atom features perform worse (Fig. 6d). They also require an accurate atom-mapping of the reaction, and we found that automatic atom mapping failed for 50 (11%) of the reactions studied. The methods above rely on expert-crafted algorithms to generate fingerprints or feature vectors. In recent years, deep learning has emerged as a method for creating such representations from the data itself, *i.e.*, representation learning. The recent reaction fingerprint from Reymond and co-workers is one such example, where the fingerprint is learned by a BERT deep learning model that is pre-trained in an unsupervised manner on reaction SMILES from patent data.^46^ Gratifyingly, the model built on the ***X***_BERT_ feature set performs on par or slightly better than ***X***_Morgan3_ with an MAE of 1.03 kcal mol^−1^ (Fig. 6d). The big advantage with the BERT fingerprint is that it does not need atom mapping and can potentially be used with noisy reaction SMILES with no clear separation between reactants, reagents, catalysts and solvents. We also used BERT fingerprints specially tuned for reaction classification to construct reaction maps, which show clear separation between different types of nucleophiles and electrophiles in the dataset (Fig. S7†). The learning curve shows that the model based on ***X***_BERT_ is more data-hungry than the models based on physical organic features, and requires *ca.* 350–400 data points to reach chemical accuracy (Fig. 8a). The radius of three for the Morgan fingerprint was chosen to incorporate long-range effects of electron-donating and electron-withdrawing groups in the *para* position of the aromatic ring (Fig. 9b). Plotting the MAE as a function of the fingerprint radius shows a clear minimum at a radius of three (Fig. 9a). With this radius, all relevant reaction information seems to be captured, and increasing the radius further probably just adds noise through bit clashes in the fingerprint generation. Encouraged by the promising results with the structural data, we wondered if a combined feature set with both the physical organic features in ***X***_full_ and the structural features in ***X***_BERT_ would perform even better. However, the model built on the combined feature set performs on par with the model built on only ***X***_full_ (Table S5†).

In summary, models based on reaction fingerprints are an attractive alternative when a sizeable dataset of at least 350 reactions are available as they are easy to develop and make very fast predictions.

### Interpretability

There has a been a push in the machine learning community in recent years to not only predict accurately but also to understand the factors behind the prediction.^50^ Models are often interpreted in terms of their feature importances, *i.e.*, how much a certain feature contributes to the prediction. Feature importances can be obtained directly from multivariate linear regression models as the regression coefficients and have been used extensively to give insight on reaction mechanisms based on ground-state features.^51^ A number of modern techniques can obtain feature importances for any machine learning technique, including SHAP values^52^ and permutation importances,^53^ potentially allowing the simple interpretation of linear models to be combined with the higher accuracy of more modern non-linear methods.

**Fig. 10:**
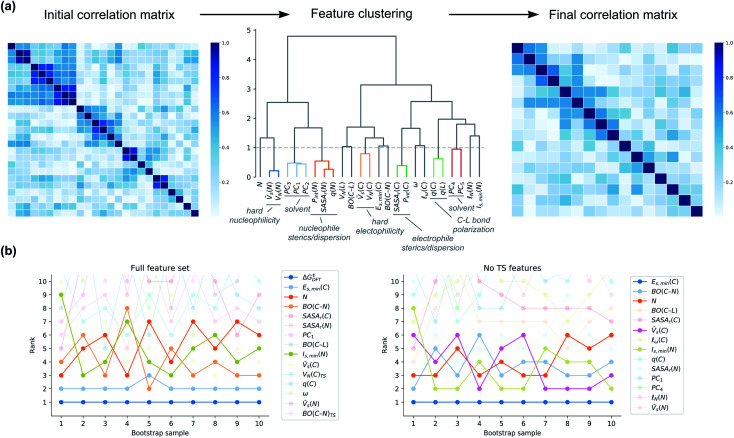
(a) Clustering of correlated features based on Spearman rank correlation. (b) Bootstrapped feature ranking for clustered ***X***_full_ and ***X***_noTS_. For identity of feature clusters, see the ESI.†

Although feature importances can be easily calculated, they are not always easily interpretable. In particular, correlation between features poses severe problems. This problem is present for our feature set, as shown by the Spearman rank correlation matrix and the variance inflation factors (VIFs)^54^ of ***X***_full_ (Fig. S8†). Therefore, special care has to be taken when calculating the feature importances, and the final interpretation of them will not be straightforward. To get around this technical problem of multicollinearity, we clustered the features based on their Spearman rank correlations, keeping only one of the features in each cluster (Fig. 10a). The stability of the feature ranking was further analysed by using ten bootstrap samples of the data.

First, we looked at how important Δ*G*^‡^_DFT_ is in our hybrid model. It turns out that it is consistently ranked as the most important feature across all bootstrap samples (Fig. 10b). The second-most important feature is the soft electrophilicity feature *E*_s,min_(C). To see more clearly which are the most important ground-state features, we analysed a model built on ***X***_noTS_ (Fig. 10b). The most important feature is again the soft electrophilicity descriptor *E*_s,min_(C). Other important features are the soft nucleophilicity descriptor *I*_s,min_(N) and the feature cluster of hard electrophilicity represented by *V̄*_s_(C). The global nucleophilicity descriptor N and the electrophile–nucleophile bond strength through BO(C–N) are also ranked consistently high.

In summary, the most important feature for the hybrid models is, as expected, the DFT-computed activation free energy Δ*G*^‡^_DFT_. Models trained without the TS features give insight into the features of substrates and nucleophiles that govern reactivity. Here, the most important features are the electrophilicity of the central carbon atom of the substrate, followed by those related to nucleophilicity. Steric and solvent features are less important. There are a number of plausible reasons why solvent features have lower importance. Firstly, the effect of the solvent is already incorporated in the Δ*G*^‡^_DFT_ through the use of both implicit and explicit solvation (for anions of second and third row elements of the periodic table). Secondly, the implicit solvent influences the values of the quantum-mechanically derived features. For the *E*_s,min_ descriptor this effect has been shown to be substantial.^34,55^ Thirdly, the solvent correction can likely be learnt implicitly also from the other features for problematic nucleophiles such as those with anionic oxygen. This aspect is also connected to the fourth factor, that solvent variation is low for the anionic nucleophiles, where for example reactions with oxygen nucleophiles are only carried out in water or methanol (Fig. S25†). There is therefore limited data for the model to learn from the solvent features for some nucleophile classes. Collection of more balanced data will be key to improved models. Steric features may become more important for other types of substrates as the current data set doesn't include very sterically crowded substrates or nucleophiles.

### Applicability domain

The applicability domain (AD) is a central concept to any QSRR model used for regulatory purposes according to the OECD guidelines.^56^ The AD is broadly defined by the OECD as “the response and chemical structure space in which the model makes predictions with a given reliability”. Although there have been many attempts to define the AD for prediction of molecular properties, there has been little work for reaction prediction models. Here, we will follow a practical approach to (1) define a set of strict rules for when the model shouldn't be applied based on the reaction type and the identity of the reactive atoms, (2) identify potentially problematic structural motifs from visualization of outliers, and (3) assess whether the uncertainty provided by the GPR_M3/2_ model can be used for risk assessment.

**Fig. 11:**
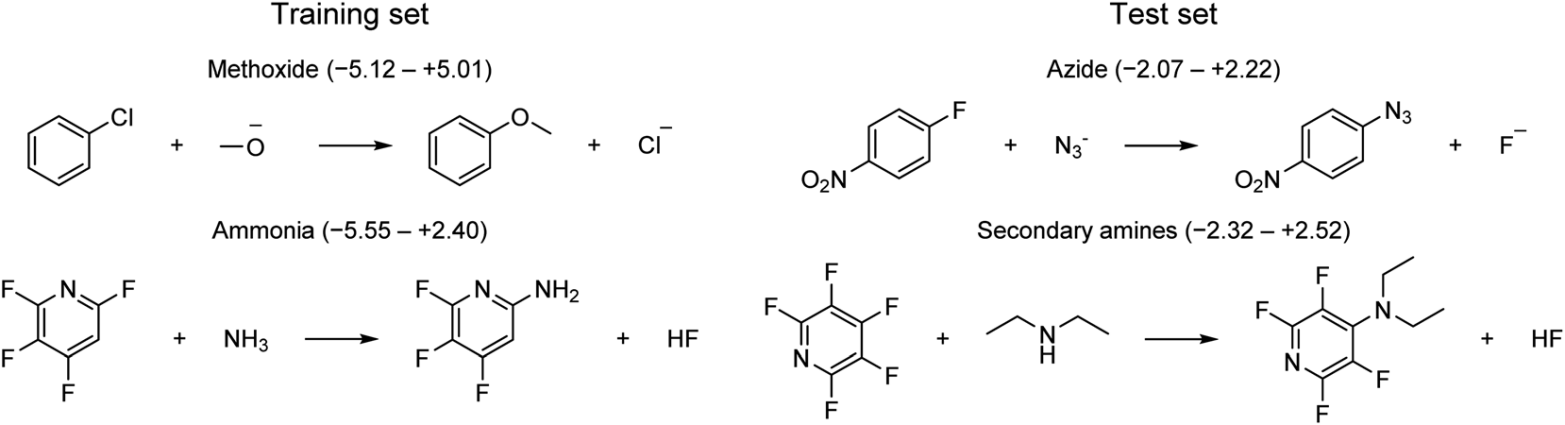
Example of outliers for training and test set. Ranges in parenthesis correspond to signed errors in kcal mol^−1^.

First, the model should only be applied to S_N_Ar reactions. Second, the model can only be used with confidence for those reactive atom types that are part of the training set (Fig. 3b). One example of reactions falling outside the applicability domain according to these rules are those with anionic carbon nucleophiles. A provisional analysis of the outliers (residual of >2 kcal mol^−1^) for the training set identifies many reactions involving methoxide nucleophile (Fig. 11). This tendency can be partly understood based on the poor performance of implicit solvation models for such small, anionic nucleophiles which is probably not corrected fully by our model. Additionally, some of these reactions with methoxide and unactivated substrates are very slow and have been run at high temperature and the rate constants have been determined through extrapolation techniques, leading to a larger error in the corresponding rates. Outliers from the test set involve azide and secondary amine nucleophiles. However, secondary amines are also the most common type of nucleophile in the dataset and are therefore expected to contribute to some outliers. For a complete list of all outliers, see the ESI.†

**Fig. 12:**
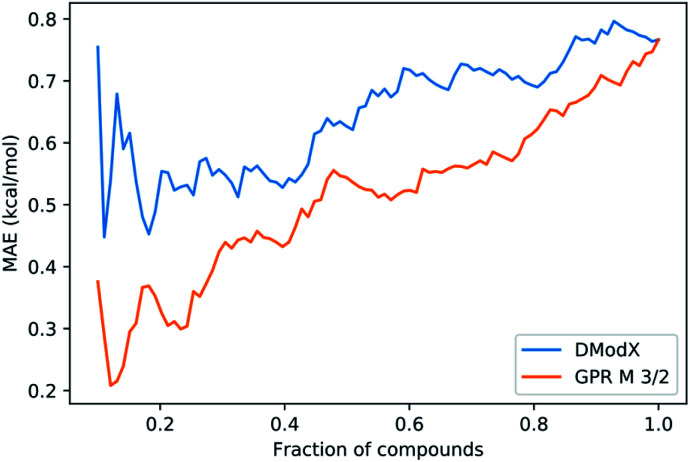
Integral accuracy averaging curve. Predicted values are ordered according to an uncertainty measure from most certain to least certain. MAE is calculated from the predictions of the GPR_M3/2_ model.

We also investigated whether the prediction uncertainty given by the GPR_M3/2_ model could be trusted. For predicting molecular properties, similarity to the training set is usually used to assess whether a prediction should be trusted (in the applicability domain) or not (outside the domain). The similarity is measured by distance metrics based on molecular fingerprints.^57^ For reactions, difference fingerprints have been shown to differentiate between reaction classes, but their suitability for defining an applicability domain within one reaction class is not clear.^58^ In the absence of a clear similarity metric, we used the Distance to Model in X-space (DModX) of a PLS model with two components (dimensions) to compare to GPR_M3/2_. PLS is a type of linear method that uses dimensionality reduction and that is used widely in chemometrics. DModX is based on distance in the latent space used by the PLS model and is an established metric for defining the applicability domain.^59^ We compared the performance of DModX to the standard deviation (std) of the GPR_M3/2_ predictions using integral accuracy averaging curves (Fig. 12). The integral accuracy averaging curve is a standard tool for evaluating uncertainty metrics, where the predicted values are ordered from most to least reliable according to the uncertainty metric.^60^ The MAE is then plotted as a function of the portion of included data. A good uncertainty metric should show a curve with an upward slope from left to right, as including more points with larger uncertainty should lead to a larger error. As can be seen from the plot, both DModX and GPR_M3/2_ std are decent measures of uncertainty. As the GPR_M3/2_ std performs best, the prediction intervals (as shown in Fig. 7) can be used directly as a measure of how reliable the prediction is.

As there are 62 reactions which occur in the dataset with more than one reaction condition (different temperature or solvent), we investigated the performance of leaving these reactions out altogether in the modelling. With this leave-one-reaction-out validation approach, we observed a MAE of 1.00 kcal mol^−1^ for GPR_M3/2_ (compared to 0.80 kcal mol^−1^ from normal cross-validation). In comparison, a model trained on ***X***_BERT_ decreased from 1.03 to 1.11 kcal mol^−1^. We also tested leave-one-electrophile-out, giving a MAE of 1.20 kcal mol^−1^ and leave-one-nucleophile-out, giving a of MAE 0.68 kcal mol^−1^. These results indicate that the model is able to predict outside its immediate chemical space with good accuracy, and not only interpolate.

Taken together, the applicability domain of our model is defined strictly in terms of the type of reaction (S_N_Ar) and the types of reactive atoms in the training set. The outlier analysis identified that extra care should be taken when interpreting the results of reactions at high temperature and with certain nucleophile classes. The width of the prediction interval from the GPR_M3/2_ model is a useful measure of the uncertainty of the prediction.

### Validation on selectivity data

To assess whether the model can be used not only for predicting rates, but also selectivities, we compiled a dataset of reactions with potential regio- or chemoselectivity issues from patent data.^61^ A total of 4365 S_N_Ar reactions were considered, of which 1214 had different reactive sites on the same aromatic ring, while only one product was recorded experimentally. A reactive site was considered as a ring carbon with a halogen substituent (F, Cl, Br and I) that could be substituted with S_N_Ar. Regioselectivity means distinguishing between reactive sites with the same type of halogen, while for chemoselectivity the halogens are different. Out of these 1214, we selected the 100 with lowest product molecular weight for a preliminary evaluation. As reaction solvent or temperature was not available, we used acetonitrile for neutral reactions and methanol for ionic reactions and a reaction temperature of 25 °C. It is possible that neglecting conditions information in this way reduces the accuracy of our model. Each possible reaction leading to different isomers was modelled by a separate predict-S_N_Ar calculation (209 in total), and the predicted major isomer was taken as the one corresponding to the lowest Δ*G*^‡^. As another testament to the robustness of the workflow, only 3 of 209 TS calculations failed (1.4%), leading finally to results for 97 reactions with selectivity issues. Of these, 66 involved regioselectivity and 31 chemoselectivity. The results show that GPR_M3/2_ was able to predict the correct site in 86% of the cases (top-1 accuracy), with 85% for regioselectivity and 87% for chemoselectivity. In comparison, predictions based on only the DFT activation energies give a comparable top-1 accuracy of 87%, with 91% for regioselectivity and 77% for chemoselectivity. It is notable that the GPR_M3/2_ model shows a much better score for chemoselectivity (87%) than DFT (77%). For regioselectivity prediction, where the same element is being substituted, DFT probably works well due to error cancellation. That the hybrid ML model performs better than DFT for chemoselectivity clearly shows that it has learnt to compensate for the systematic errors in the DFT calculations. In fact, it seems that the ML model is balancing the gain in chemoselectivity prediction with a loss in regioselectivity (85%) compared to DFT (91%), leading to a comparable accuracy on both tasks. More training data is expected to alleviate this loss in regioselectivity accuracy. Also, inclusion of the actual solvents and temperatures could also increase accuracy. Further validation work could extract the conditions from the original patents, or use a dataset that already contains this information.

Overall, it is remarkable that the hybrid model GPR_M3/2_ performs so well (86%) for selectivity as it was not explicitly trained on this task. In comparison, for electrophilic aromatic substitution (S_E_Ar), single-task deep learning models trained for selectivity prediction of bromination, chlorination, nitration and sulfonylation achieved top-1 accuracies of 50–87%.^62^ For bromination, the RegioSQM model based on semiempirical energies of the regioisomeric intermediate σ-complexes achieved 80%, compared to 85% for the neural network just mentioned. In light of these models, the 86% top-1 accuracy obtained with GPR_M3/2_ for S_N_Ar looks very competitive. Likely, hybrid models explicitly trained for selectivity prediction can perform even better. Another approach would be to use transfer learning to repurpose deep learning models for barrier prediction to selectivity prediction.

## Conclusions and outlook

We have created hybrid mechanistic/machine learning models for the S_N_Ar reaction which incorporate TS features in addition to the traditional physical organic features of reactants and products. The chosen Gaussian Process Regression model achieved a mean absolute error of only 0.77 kcal mol^−1^ on the external test set, well below the targeted chemical accuracy of 1 kcal mol^−1^. Furthermore, the model achieves a top-1 accuracy for regio- and chemoselectivity prediction on patent reaction data of 86%, without being explicitly trained for this task. Finally, the model comes with a clear applicability domain specification and prediction error bars that enables the end user to make a contextualized risk assessment depending on what accuracy is required.

**Fig. 13:**
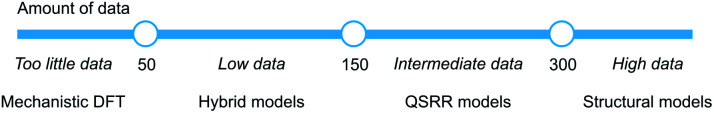
Models for quantitative rate prediction for different data regimes based on the S_N_Ar dataset.

By studying models built on reduced sets of the physical organic features, as well as reaction fingerprints, we identified separate data regimes for modelling the S_N_Ar reaction (Fig. 13). In the range 0–50 samples, it is questionable whether accurate and generalizable machine learning models can be constructed.^63^ Instead, we suggest that traditional mechanistic modelling with DFT should be used, with appropriate consideration of its weaknesses. With 50–150 samples, hybrid models are likely the most accurate choice, and should be used if time and resources for their development is available. In the range 150–300 samples, traditional QSRR models based on physical organic features reach similar accuracy as hybrid models, while models based on purely structural information become competitive with over 300 samples. It will be very interesting to see if these numbers generalize to other reaction classes. For choosing features for QSRR models, it is notable that electronic reactivity features from DFT were consistently ranked high in the feature importances, and we saw that the inclusion of surface features makes the models significantly better.

Our workflow can handle the mechanistic spectrum of concerted and stepwise S_N_Ar reactions, and we are currently working on extending it to handle the influence of general or specific acid and base catalysts as well as treating related reaction classes. This general model is a significant improvement in generality compared to previous work, which modelled selectivity of S_N_Ar reactions in terms of the relative stability of the σ addition complexes and therefore was only applicable to reactions with step-wise mechanisms.^64–66^ Although promising, we believe that widespread use of hybrid models is currently held back by difficulties in computing transition states in an effective and reliable way. We envision that this problem will be solved in the near future by deep learning approaches that can predict both TS geometries^67^ and DFT-computed barriers^27^ based on large, publicly available datasets.^68,69^ In the end, machine learning for reaction prediction needs to reproduce experiment, and transfer learning will probably be key to utilizing small high-quality kinetic datasets together with large amounts of computationally generated data. Regardless of their construction, accurate reaction prediction models will be key components of accelerated route design, reaction optimization and process design enabling the delivery of medicines to patients faster and with reduced costs.

## Conflicts of interest

There are no conflicts to declare.

## Supplementary Material

SC-012-D0SC04896H-s001

SC-012-D0SC04896H-s002
