# Supplementary material for: Machine learning meets mechanistic modelling for accurate prediction of experimental activation energies
Source: Chem Sci. 2020 Nov 5;12(3):1163–75. doi: 10.1039/d0sc04896h (PMC9528810; doi:10.1039/d0sc04896h)
Supplement: SC-012-D0SC04896H-s001 [file SC-012-D0SC04896H-s001.pdf]

Electronic Supplementary Material (ESI) for Chemical Science.
This journal is © The Royal Society of Chemistry 2020

Supporting Information

1 Contents

1 Contents 1
2 Predict-S_NAr workflow 2
2.1 Input preparation 2
2.2 Input parsing and structure generation 3
2.3 Mechanistic calculations 3
2.3.1 Quantum chemistry 3
2.3.2 Transition state calculations 4
2.3.3 Use of additional constraints for *xtb* optimizations 5
2.3.4 Calculation monitor 6
2.3.5 Explicit solvation 6
2.3.6 Use of electronic temperature in *xtb* calculations 8
2.4 Feature generation 8
2.5 Running the workflow 10
3 Machine learning 11
3.1 Model selection 11
3.1.1 Models tested 14
3.2 Dataset visualization 15
3.3 Feature importances 15
3.4 Learning curves 20
3.5 Analysis of reactions with large errors 20
3.6 Dependence on number of heavy atoms 22
3.7 Y-randomization 23
4 Regio- and chemoselectivity validation 23
5 Experimental dataset 24
5.1 Description of the modelling data 24
5.2 Distribution of activation free energies 26
5.3 Most common substrates and nucleophiles 26
5.4 Replicated reactions 27
5.5 Conditions 27
5.6 Reaction temperatures 28
5.7 Solvent distribution 29
6 Workflow database 29
7 Analysis package 31

8 References 31

2 Predict-S_NAr workflow

The *predict-S_NAr* workflow takes a reaction SMILES[1] as input, identifies the nucleophile, substrate, product and leaving group and then sets up and performs all calculations of the mechanism and the descriptors (Figure S1). Temperature and solvent are also used throughout the calculations. The results are stored in a database for easy retrieval. *predict-S_NAr* has been optimized for robustness and includes extensive error checks for the quantum chemical calculations. Below, a detailed description of the different steps will be given.

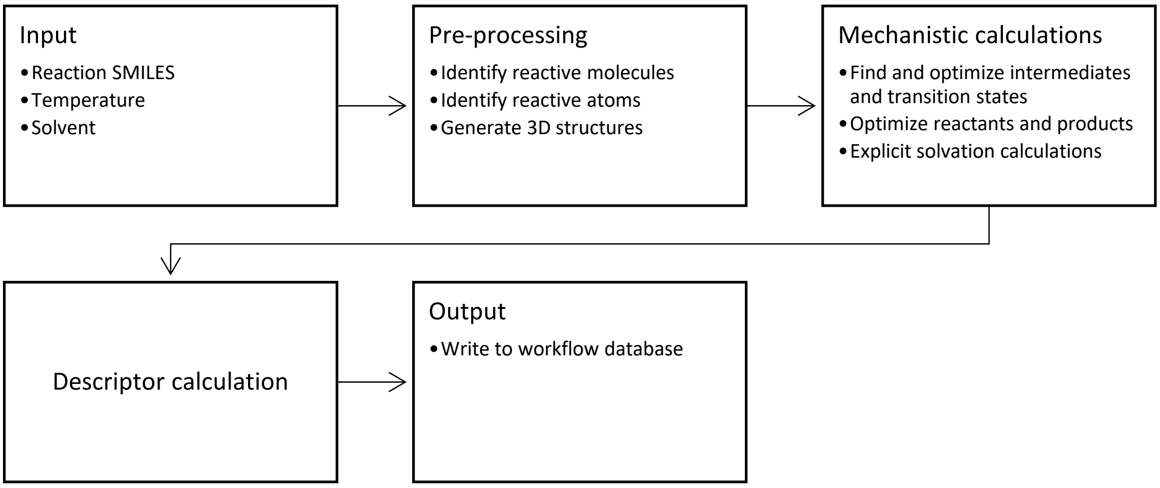

Figure S1. Overview of *predict-S_NAr* workflow

2.1 Input preparation

The literature data from the file "kinetic_data_v4.xlsx" was first pruned to remove entries for which either activation free energy, solvent or temperature was missing. (After the modelling was completed, some additional entries have been added in database, see section 5.)

Solvent mixtures were processed to determine the "influential solvent" as the SMD solvent model (*vide infra*) can only handle single solvents. It is clear that solvent properties are not a simple linear interpolation between the properties of the constituent solvents. Determining which single solvent to substitute for a solvent mixture is somewhat arbitrary, but we used two principles to guide our reasoning: (1) preferential solvation and (2) activity.[2] Preferential solvation means that ions will be preferentially solvated by the solvent to which they have the strongest interactions. More polar solvents should therefore have a larger influence in solvating ionic reactants than expected based on their molar fraction in comparison with less polar solvents. Activity coefficients will be higher for minority solvents, meaning that they will exert a higher "effective" mol fraction than the raw numbers indicate. By combining these two principles, we came up with the following rule of thumb for binary solvent mixtures: if the polar solvent has a mole fraction of at least 0.2, it will be used as the single solvent in the workflow, otherwise the less polar solvent will be used.

The input preparation process is documented in the Jupyter notebook “prepare_kinetic.ipynb” in the folder “prepare_kinetic_data”. The solvent is parsed with *SolventPicker* object, where the influential solvent is selected. The work to generate the solvent data is given in the Jupyter notebook “Solvents.ipynb” in the folder “solvents”.

2.2 Input parsing and structure generation

The input to the *predict-S$_N$Ar* workflow is the reaction SMILES, the reaction temperature in Kelvin and the solvent name. The solvent is converted to SMILES via the Open Parser for Systematic IUPAC nomenclature (*OPSIN*) tool (2.4.0).[3,4] It is then checked against a list of solvents available in the *Gaussian16* program. If an exact match cannot be found, the most similar solvent based on distance in the standardized three-dimensional space of dielectric constant (ε) and hydrogen bonding properties (Abraham’s AH and BH) is used.[5] If the hydrogen bonding parameters are not available for the input solvent, the choice is based on just the dielectric constant. The same process is used for the *xtb* program, which has a much more limited selection of solvents. Note that *xtb* energies are only used in intermediate steps of the workflow and do not appear in the final output of the model. Therefore it does not matter significantly what solvent is chosen for *xtb* step, as long as the right structures are found and later optimized with *Gaussian16*.

The reaction SMILES is parsed first by the *ReactionSmilesParser* object which identifies substrate, nucleophile, product and leaving group based on minimum common substructure matches (with some additional rules pertaining specifically to the S$_N$Ar reaction). If the leaving group is not specified, it is created. Intramolecular reactions are identified. Molecules which do not take part in the bond breaking and bond making events are sorted as agents and placed “above the arrow” in the reaction SMILES (in between the two “>>”). An *AgentDetector* object parses the agents to find acids and bases. The reactive atoms are also identified.

The *Smiles2XYZ* object uses the output from the *ReactionSmilesProcessor* to construct 3D structures of all reactants and products using the *RDKit*.[6] The conformer generation recipe of Deane and co-workers is used,[7] with the ETKDG algorithm by Landrum and Riniker.[8] Structures are optimized and ranked with the MMFF[9,10] or UFF[11] force fields, depending on atom availability, keeping only the lowest-energy conformer. *Smiles2XYZ* also constructs a reaction complex with the nucleophile situated at a distance of 6 Å from the substrate to serve as a starting point for further calculations with *xtb*. *Smiles2XYZ* further identifies the atoms of the reactive ring and if the nucleophile and transition state would be candidates for implicit/explicit solvation modelling (Section 2.3.5). The criterion for explicit solvation is that the nucleophilic atom should be a negatively charged and of the second or third row of the periodic table. The rationale is that these small anions should be more localized and harder for the implicit solvation models to treat. *Smiles2XYZ* also detects the following cases which are of interest in the further modelling:

1. Azide nucleophiles
2. *Ortho* nitro groups on the substrate
3. Non-conjugated nucleophilic atoms (adjacent to *sp*^{3}-hybridized atom)

2.3 Mechanistic calculations

2.3.1 Quantum chemistry

DFT calculations used Gaussian 16 Rev C.01.[12] Geometry optimizations employed the ωB97X-D functional[13] with the 6-31+G(d)[14,15] basis set as this functional has shown good performance for S$_N$Ar reactions.[16] All stationary points were confirmed with frequency calculations. Thermal contributions to the free energies were corrected for low-frequency modes with Grimme’s quasi-harmonic scheme[17]

using GoodVibes 3.0.1[18,19] at the reaction temperature. Final energies were obtained by combining single-point energies with ωB97X-D/6-311+G(d,p)[20] with the thermal free energies at the ωB97X-D/6-31+G(d) level. Solvent effects were accounted for by the SMD solvation model.[21] GFN2-xTB[22] calculations were performed with the *xtb* 6.2 software.[23] Solvent effects with *xtb* were treated with the GBSA solvation model. To better model anions, *xtb* calculations employed an electronic temperature (2000–7000 K) to simulate the effect of diffuse basis functions (Section 2.3.6). Solvation of anionic nucleophiles are generally treated poorly by continuum models, especially in polar solvents. To correct our solvation energies, we used an automatized approximate version of the cluster-continuum model of Pliego and Riveros (Section 2.3.5).[24,25]. For all structures, we performed conformational sampling with *CREST* (2.8)[26] and *xtb*. The resulting conformers were ranked according to their electronic energy with ωB97X-D/6-31+G(d) and only the lowest energy conformer was selected for further optimization.

2.3.2 Transition state calculations

We first checked whether we could locate a stable σ complex to assess whether the reaction was concerted or stepwise. If the σ complex was stable, we scanned each reactive bond separately using *xtb*, starting from the σ complex, to find the TSs for addition and elimination. If the σ complex was not stable, we performed a concerted scan of the reactive bonds using generalized internal coordinates (GICs) to located the concerted TS, scanning from the reactive complex to the product complex. Single point DFT calculations with ωB97X-D/6-31+G(d) along the scan coordinate identified the approximate location of the TS, which was conformationally sampled with frozen reaction core and subsequently fully optimized with DFT. TS conformations were sampled with atom position constraints on the aromatic core and bond constraints for the reactive bonds. The reactions involving aliphatic alkoxides as leaving groups had unrealistically large activation energies owing to proton-transfer being involved in the rate-determining step. Our workflow treats this proton transfer as going from the nitrogen to the oxygen in a concerted manner, while in reality it would occur by two separate proton transfers involving the solvent.[27] Owing to the inadequacy of our model, we use the barrier from the addition step as rate-determining in the later machine learning, in accordance with the literature.[28] Proper treatment of these types of mechanisms will be the topic of a future study.

Reactant and product complexes were optimized with GFN2-xTB at a C–Nu distance corresponding to the sum of the vdW radii of the two reactive atoms. The distance to the *ortho* carbons were also frozen at distances determined from the C–Nu and C–*ortho* C distances together with the Pythagorean theorem. For intermediate optimizations, the *xtb* optimization and *CREST* conformational search used frozen C–LG and C–Nu bond lengths which were taken from the GFN2-xTB geometries of the substrate and the product, respectively. For finding transition states, the GFN2-xTB energy profile was analysed with respect to peaks higher than 0.01 kcal/mol, which were identified as candidate transition states. A bond order criterion discarded peaks for which the bond order of any of the reactive bonds changed by less than 0.05 Å. If no peak could be identified, the workflow moved on to the next stage, except in the case of the second step of a stepwise mechanism, where the first geometry on the bond scan was taken as a TS guess. The rationale was that the barrier could be very small and the peak had probably been missed by the bond scan procedure. The peaks were sorted with respect to energy and an attempt to locate the TS from the geometry of the first peak was attempted. If a TS could not be found, the program proceeded to the next peak in the list. In the special case where concerted bond breaking and proton transfer could occur in the second step of a stepwise mechanism, a GIC scan was conducted to find additional guess transition states of this type if no other TS could be found. Transition states were validated by projecting the normalized Cartesian coordinate displacements from Gaussian16 of the TS mode onto the reactive bonds. Any TS candidate with a projected displacement below 0.13 units were rejected. TS structures were optimized by DFT after the initial CREST conformational search as described

above. First, the geometry was optimized with the reactive bonds frozen, and then a full TS optimization was conducted.

For molecules including iodine, we used an effective core potential (ECP). The solution was to combine the def2-SVPD and def2-TZVPD basis sets[29,30] and their associated ECPs together with the 6-31+G(d) and 6-311+G(d,p) basis sets for the rest of the atoms, for double and triple zeta basis set calculations, respectively. Generation of the basis set dictionaries is documented in the Jupyter notebook “create_pickle_files.ipynb” in the directory “basis sets”.

2.3.3 Use of additional constraints for *xtb* optimizations

We used some additional constraints for *xtb* calculations to avoid complications were *xtb* would favour geometries that would be very high in energy at the DFT level or lead to discontinuities in bond scans. These constraints were lifted for the final DFT optimization and do not impact the final geometries that are used to calculate the reaction barriers and the descriptors.

We found that the combination of *ortho* nitro groups on the substrate together with amine nucleophiles could cause problems with *xtb* optimizations using an electronic temperature. During optimizations and conformational sampling of intermediates and transition states, spurious proton transfer could happen from the amine to the nitro group. Therefore, we froze the N–H bonds for this type of calculations to prevent the spurious transfer.

Azide nucleophiles tended to bend at the GFN2-xTB level in intermediates and transition states, which could cause problems with discontinuities in the DFT single point calculations along the GFN2-xTB bond scans. We therefore imposed two constraints for these cases (Figure S2):

1. The N–N–N angle is frozen to 180 degrees (between atoms 15, 16 and 17 in the figure)
2. The Lg–C–N–N dihedral angle is frozen at 180 degrees (between atoms 10, 3, 17 and 16 in the figure).

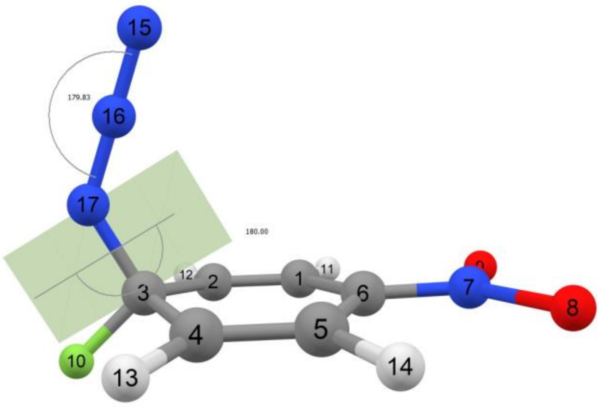

Figure S2. Constraints for azides used in *xtb* optimizations and conformational samplings. Angle constraint of 180 degrees for the angle 15-16-17 and a dihedral angle constraint of 180 degrees for 10-3-17-16.

For bond scans, we also implemented an additional set of constraints. For scans, for the first step of a step-wise mechanism, we implemented constraints for negatively charged oxygen nucleophiles which were connected to saturated atoms. Due to lack of diffuse functions, the O–C bond is artificially short with GFN2-xTB due to negative hyperconjugation effects to relieve the instability of the negative charge on the oxygen atom. We therefore froze the C–O distance during the scan to the value calculated with DFT for the isolated nucleophile.

For all the GIC bond scans, we also froze the difference of the distances between the nucleophilic atom and the *ortho* carbons of the substrate to ensure a symmetric approach of the nucleophile. For the bond scans from the intermediate with *xtb*, we instead scanned the Nu–*ortho* C distances together with the C–Nu distance.

2.3.4 Calculation monitor

The calculation monitor handles common errors and issues that occur during electronic structure calculations and geometry optimizations.

Displacement of negative frequencies for geometry optimizations
If a minimum structure is optimized and the frequency calculation shows one or more negative frequency, the structure was displaced along the corresponding normal mode and the optimization restarted. For the workflow, one such preoptimization was done.

Dissociation check
The calculation monitor checked for dissociation of bonds in the optimization of the intermediate. Dissociation was judged to have occurred if the bond orders of either the C–Nu or C–Lg bond decrease below 0.3. The calculation was then aborted and the workflow proceeded to calculate a concerted mechanism.

Adaptive keyword changes in response to SCF errors and coordinate system errors
Common convergence errors are captured and appropriate Gaussian keywords are inserted to try to remedy the situation. One example is the error "Convergence failure -- run terminated" for which the keyword “scf=xqc” is used. Coordinate system errors are also addressed, *e.g.*, “RedCar failed” which is addressed with a series of Gaussian IOps: "1/59=10", "1/59=14", "1/59=4", "1/59=40" and "1/59=44". Also more obscure and non-reproducible errors such as “Internal input file was deleted!” are handled by simply restarting the calculation.

2.3.5 Explicit solvation

Explicit solvation is handled via the cluster-continuum model of Rivero and Pliegos.[25] We targeted the energy that is missed by the implicit solvation model, which can be corrected by using explicit solvent molecules. We denote the free energy in solvent according the full cluster-continuum model with *n* explicit solvent molecules as $\Delta G_{solv,n}(solute)$, while that using the implicit model is $\Delta G_{solv}(solute)$. The correction to the implicit model is then given by

$$\Delta G_{solv,n}(solute) - \Delta G_{solv}(solute) = \Delta G_{solv}(cluster) - \Delta G_{solv}(solute) - n\Delta G_{solv}(solvent) \quad \text{Equation 1}$$

where $\Delta G_{solv}(cluster)$ is the free energy of the cluster, $\Delta G_{solv}(solute)$ the free energy of the solute and $\Delta G_{solv}(solvent)$ is the free energy of the solvent (with the appropriate standard state), using the implicit solvation model. The number of solvent molecules, *n*, is determined by a variational principle, where the *n* which gives the largest correction is the most appropriate. This variational principle stems from two competing factors. The first factor is the stabilizing effect due to specific interactions between the explicit solvent molecules and the solute, which are not captured fully by the implicit solvation model. The second factor is the destabilizing effect of bringing solvent molecules from the bulk solvent to form the cluster, which corresponds to an entropic loss.

We have implemented an approximate version of the cluster-continuum model that uses a combination of GFN2-xTB and DFT to select the optimal number of solvent molecules (Figure S3). The final correction term is then calculated with full DFT.

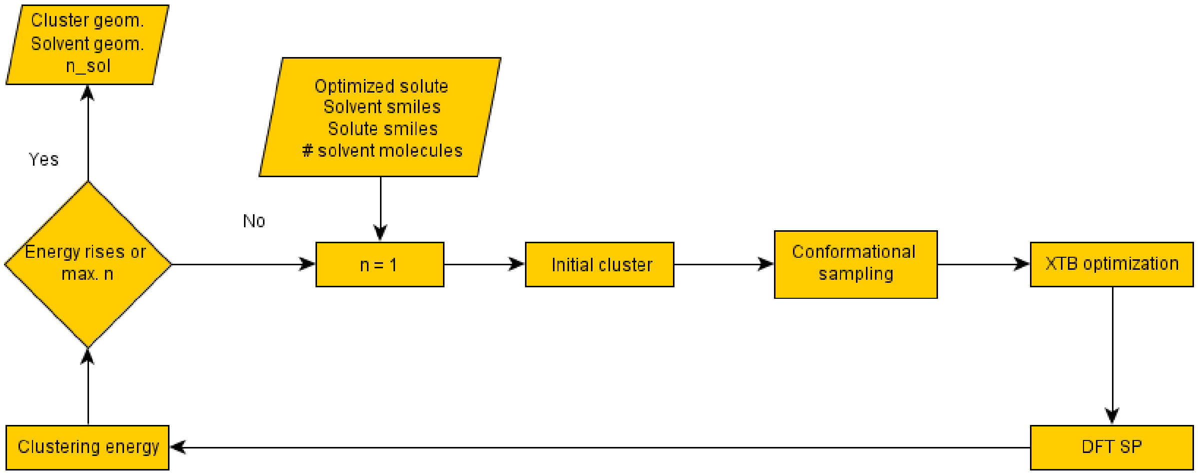

Figure S3. Sketch of automated cluster generation process.

The first step of the procedure is to generate a cluster geometry. We start by placing solvent molecules around the solute at a distance of 4 Å at the geometries given in Table S1.

Table S1. Geometry of initial placement of solvent molecules.

n	Geometry
1	Point
2	Line
3	Equilateral triangle
4	Tetrahedron
5	Trigonal bipyramid
6	Octahedron

The cluster is the optimized with a small force constant (0.0005) pulling the solvent molecule towards the closest atom in the molecule, to form a crude guess for the cluster geometry. The pulling step is followed by two rounds of CREST conformational searches in the non-covalent interactions (NCI) mode. The conformers generated in the second run are ranked with DFT, and cluster free energy is constructed from the DFT single-point energy together with the free-energy contributions from GFN2-xTB. The energy of the solvent and solute are calculated in the corresponding manner, and the solvent correction term is calculated from Equation 1. Solvent molecules are added iteratively until the solvent correction terms decreases in magnitude according to the variational principle. A cut off of 2.0 kcal/mol is used to decide whether to go ahead with the final DFT cluster optimization. Special treatment is done for hydroxide in water, where GFN2-xTB optimization gives a delocalized structure where the proton is shared equally between two O atoms (

Figure **S4**). In this case, the O–H bond lengths are frozen for all steps. For transition states, the aromatic core and the reactive atoms are frozen. For the final DFT optimization of the cluster at the optimized number of solvent molecules, all constraints are released. For transition states, this means a full TS optimization for the cluster. The final solvent correction at the DFT level is then again calculated according to Equation 1.

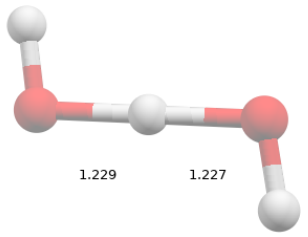

Figure S4. Hydroxide with one explicit water molecule optimized with GFN2-xTB

2.3.6 Use of electronic temperature in *xtb* calculations

The *xtb* program uses a standard electronic temperature of 300 K. We found that a higher electronic temperature could serve as a substitute for diffuse functions for the GFN2-xTB method, which lacks them by default. The rationale is that electrons of the approaching anionic nucleophile could partly delocalize into the π* orbitals of the aromatic ring. However, if the delocalization becomes too strong, complete charge transfer is the result. It is therefore necessary to regulate the electronic temperature carefully to keep in the window where the lack of diffuse functions is remedied, while the problems of charge transfer don't become too apparent. We found that a good starting guess could be based on the energy gap between the HOMO energy of the nucleophile and the LUMO energy of the substrate. The following rules were used to set the initial electronic temperature, which could later be changed for some steps.

HOMO-LUMO gap (eV)	Electronic temperature (K)
(−∞) – (−0.5)	4000
(−0.5) – (+∞)	7000

After the initial setting based on the separated reactants, the following rules were used, which reflect that the HOMO-LUMO gap of the reaction complex is different than for the separated reactants.

HOMO-LUMO gap (eV)	Electronic temperature (K)
(−∞) – (+2.0)	4000
(+2.0) – (+∞)	7000

Bond scans and conformational scans are examples were the workflow will recheck the HOMO-LUMO gap and adjust the electronic temperature. For bond scans with neutral nucleophiles, the following ranges are used.

HOMO-LUMO gap (eV)	Electronic temperature (K)
(−∞) – (+1.0)	2000
(+1.0) – (+2.0)	4000
(+2.0) – (+∞)	7000

2.4 Feature generation

The solvent-accessible surface area and the P_{int} dispersion descriptor were calculated with an in-house development version of the *morfeus* software.[31] The SASA calculation used the algorithm by Shrake and Rupley[32] with the vdW atomic radii taken from the CRC Handbook.[33] P_{int} was calculated on a surface constructed from the atomic radii of Rahm and co-workers[34] and with the D3 dispersion coefficients.[35]

Electronic structure features were calculated based on B3LYP/6-31+G(d) calculations with the SMD solvation model. The local electron attachment energy[36] and the average local ionization energy[37] as well as the surface electrostatic potential were calculated with the *HS95* program (version 190510).[38] The $I_{s,min}$ and $E_{s,min}$ values were taken as the lowest values on the atomic surface, regardless if there was a stationary point associated with the atom in *HS95*. The five solvent PCA components compiled by Diorazio *et al.* were used as solvent features.[5] DDEC6 charges and bond orders were calculated with

Chargemol (3.5)[39,40] The electrostatic potential at the nuclei (V_N) was calculated with Gaussian 16 using the keyword *prop=potential*. The global nucleophilicity parameter N was calculated as the negative of the ionization potential of the substrate in solution.[41] The global electrophilicity parameter ω was calculated as

$$\omega = \frac{\mu^2}{2\eta} \quad \text{Equation S2}$$

where η is the chemical hardness given by

$$\eta = IP - EA \quad \text{Equation S3}$$

Here, IP and EA are the vertical ionization potential and electron affinity, respectively, and μ is the chemical potential,[42] given by

$$\mu = -\frac{IP + EA}{2} \quad \text{Equation S4}$$

The local electrophilicity descriptor was calculated as

$$l_\omega = -\frac{\mu}{\eta} f + \frac{1}{2}\left(\frac{\mu}{\eta}\right)^2 f^2 \quad \text{Equation S5}$$

where f is the quadratic Fukui function, and f^2 is the dual descriptor.[43] The local nucleophilicity descriptor was calculated as

$$l_N = f^- \quad \text{Equation S6}$$

where f^- is the Fukui function for electrophilic attack. The quadratic Fukui function was calculated as

$$\frac{q^- + q^+}{2} \quad \text{Equation S7}$$

where q^- is the charge of the atom in the anion, and q^+ is the charge of the atom in the cation. The dual descriptor was calculated as

$$f^2 = f^+ + f^- \quad \text{Equation S8}$$

where f^+ is the Fukui function for nucleophilic attack. The Fukui functions for nucleophilic and electrophilic attack were in turn calculated as

$$f^+ = q - q^+ \quad \text{Equation S9}$$

and

$$f^- = q^- - q \quad \text{Equation S10}$$

where q is the charge of the neutral molecule. We used atomic Hirshfeld charges[44] that were calculated with Gaussian 16 using the *population=hirshfeld* keyword.

Morgan fingerprints were calculated with the *RDKit* (2020.03.1.0)[6] as count vectors and length 1024 bits. Reaction difference fingerprints were obtained by subtracting the summed fingerprints of the reactants from the summed fingerprints of the products. Using bits instead of counts, or using a length of 512 or 2048 instead 1024 did not change the results significantly (Table S5).

Reactions were atom-mapped using *Biovia Pipeline Pilot 2018* (18.1.0.1604),[45] and erroneous mappings were corrected by hand using *ChemFinder* (19.0.0.22) and *ChemDraw* (19.0.0.22). CGRs were generated

with *CGRtools* (4.0.18)[46,47] and were aromatized and standardized. ISIDA descriptors were then obtained with *CIMtools* (4.0.2)[48] and *Fragmentor 2017*. Sequence features were generated with the following keywords: *"fragment_type=3"*, *"min_length=2"*, *"cgr_dynbonds=1"*, *"doallways=True"*, *"useformalcharge=True"* and *"max_length=6"* or *"max_length=8"* was used. Atom features were generated with the following keywords: *"fragment_type=6"*, *"min_length=2"*, *"cgr_dynbonds=1"*, *"useformalcharge=True"* and *"max_length=4"* or *"max_length=8"* was used. The generation of the descriptors is documented in the Jupyter notebook "cgr_tools.ipynb" in the folder "cgr_isida_descriptors". One-hot encoded (OHE) features were created using the full data set prior to the train-test split. The encoding was based on the identity of the substrate, nucleophile, product, leaving group and solvent, as given by their InChIKeys. The BERT reaction fingerprints[49] (of fixed length 256 bits) were generated with the *rxnfp* (0.0.1) package.[50] For prediction, we used the pre-trained model ("bert_pretrained"), and for making reaction maps we used the fine-tuned model ("bert_ft"). The generation of the BERT fingerprints is documented in the Jupyter notebook "rxnfp.ipynb" in the folder "bert_rxnfp". The structural feature sets as well as the OHE feature set was amended with the PC_1–PC_5 solvent descriptors.

2.5 Running the workflow

The workflow is designed as a Python package `predict_snar`, version 0.1.0, that can be installed with "`pip install .`". The Python package requirements are specified in the "setup.py" file and listed in Table S2.

Table S2. Python packages required to run the *predict-S_NAr* workflow.

Requirement	Used version	Description
ase	3.18.0	Atomic simulation environment.[51] Handling of structure information.
cclib	1.6.2	Parsing of quantum-chemical chemistry output.[52] Documentation.
joblib	0.13.2	Parallelization of calculations. Documentation.
mendeleev	0.4.5	Periodic table information such as covalent radii and vdW radii. Documentation.
numpy	1.16.4	Array manipulation and mathematical calculations. Documentation.
goodvibes	3.0.1	Corrections to quantum-chemical free energies. Documentation.
scipy	1.3.2	Constants and unit conversion. Distance calculations. Peak finding.
steriplus	0.3.0	Calculation of P_{int} and $SASA_r$ descriptors. Available in the near future as *morfeus*.

The workflow is designed for running on a Linux cluster. Required software is listed in Table S3.

Table S3. Software required to run the *predict-S_NAr* workflow.

Software	Description
Gaussian16	Quantum-chemistry. Commercial license.
HS95	Electronic descriptors. Needs permission from Tore Brinck
Chargemol	Charge and bond order descriptors. Install from here.
interface_script	A Python script to handle communication between *xtb* and Gaussian (included)
xtb	Semi-empirical quantum chemistry. Install from here.
CREST	Conformational sampling. Install from here.

The workflow has been tested with the versions of software noted in Section 2.3. A configuration file called ".ps_config" must be placed in the user home folder with desired default options and directories to software. Java must also be available in the environment to run the packaged OPSIN tool.

```
[DFT]

solvation_model = smd

sp_solvation_model = smd

nosymm = True
```

```
[DIRECTORIES]
xtb = /projects/cp/programs/xtb/default # Path to xtb executable
crest = /projects/cp/programs/crest/default # Path to crest executable
chargemol = /projects/cp/programs/chargemol/default # Path to chargemol executable
hs95 = /projects/cp/programs/hs95/default # Path to hs95 executable
interface_script = /projects/cp/programs/scripts/predict-snar/interface-g16-xtb # Path to interface script executable
crest_scratch = /dev/shm/ksrf385 # Scratch directory for CREST program
gaussian_scratch = /scratch/ksrf385 # Scratch directory for Gaussian program
atomic_densities = /projects/cp/programs/chargemol/default/atomic_densities/ # Directory to atomic densities used by the chargemol program.
```

A config file must be created in the directory before running the workflow. This is done by running the script `ps_create_config`. For instructions on its use, run `ps_create_config --help`.

The workflow is invoked with the command

```
python -m  predict_snar -p <n_cpus> -m <mem in GB> '<reaction SMILES>'
```

where the argument "-p" specifies the number of CPUs to use and "-m" the amount of available memory in GB. An example job script is included in the supporting information with the article. Two example outputs from the workflow are also given.

3 Machine learning

The machine learning is reproduced in the Jupyter notebooks listed in Table S4.

Table S4. Jupyter notebooks used for machine learning.

Notebook file	Description
"train_test_split.ipynb"	Data pre-processing, feature generation, train-test split.
"modelling.ipynb"	Model selection
"testing.ipynb"	Validation on external test set. Dataset visualization. Feature importances. Learning curves.

3.1 Model selection

For the model building, we used the Python machine learning package *scikit-learn* (0.22.1).[53] Data was handled by the *pandas* (1.0.3) data analysis and manipulation package.[54] Plots were generated with *matplotlib* (3.1.0)[55] and *seaborn* (0.9.0).[56] We first processed the dataset in accordance with the description in Section 5.1 and put the reactions through the workflow(Section 2). We then split the dataset randomly into a training set (80%) and a test set (20%) and proceeded with model selection on the training set. As the first step in the model selection, we choose between different variations of the DFT activation energies, seeing that the best performer was with full cluster-continuum solvent treatment of both nucleophile and TS, using the Grimme quasi-harmonic correction for the free energies (Figure S5). We investigated correlation between the features using the Pearson[57] correlation matrix and the variance inflation factors.[58] We confirmed the initial feasibility of modelling using linear regression and inspecting the normal probability plot (Q-Q plot) of the residuals, with reassuring result.

(a) Cluster-continuum solvation of nucleophile and TS

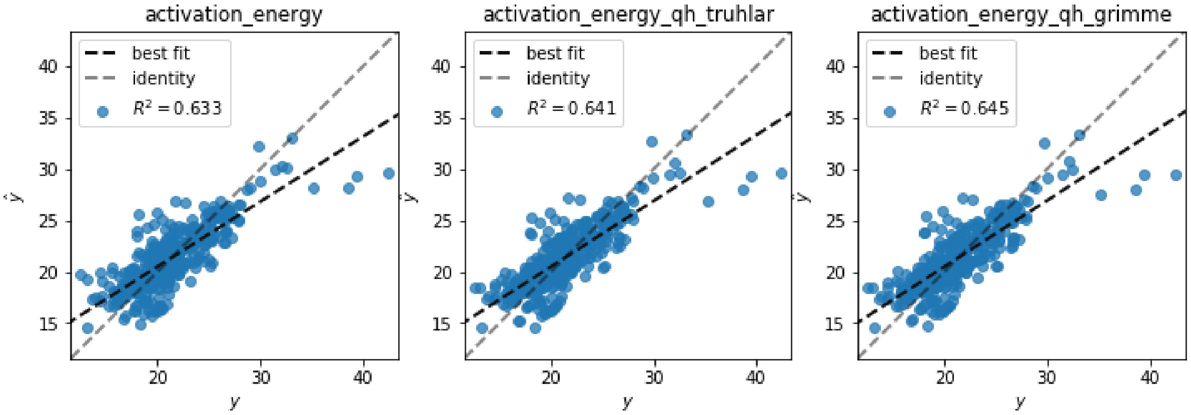

(b) Cluster-continuum solvation of nucleophile

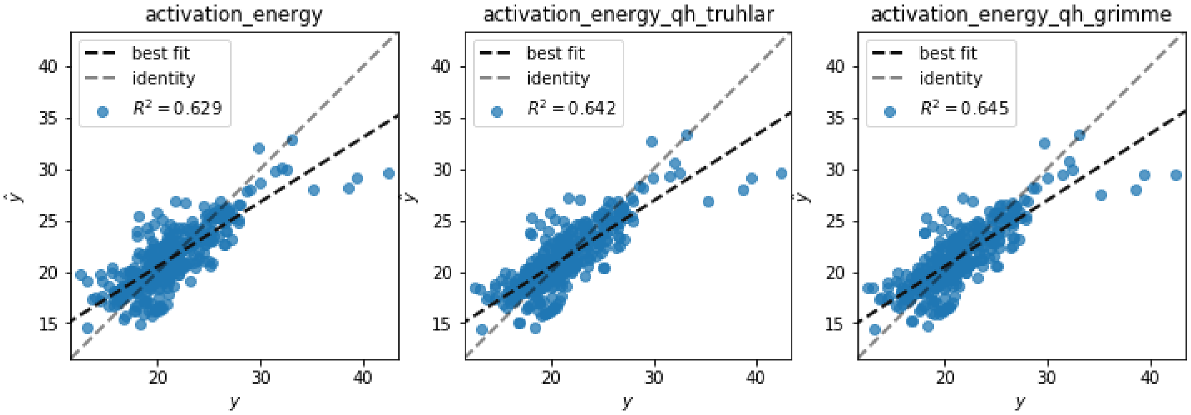

(c) No cluster-continuum solvation

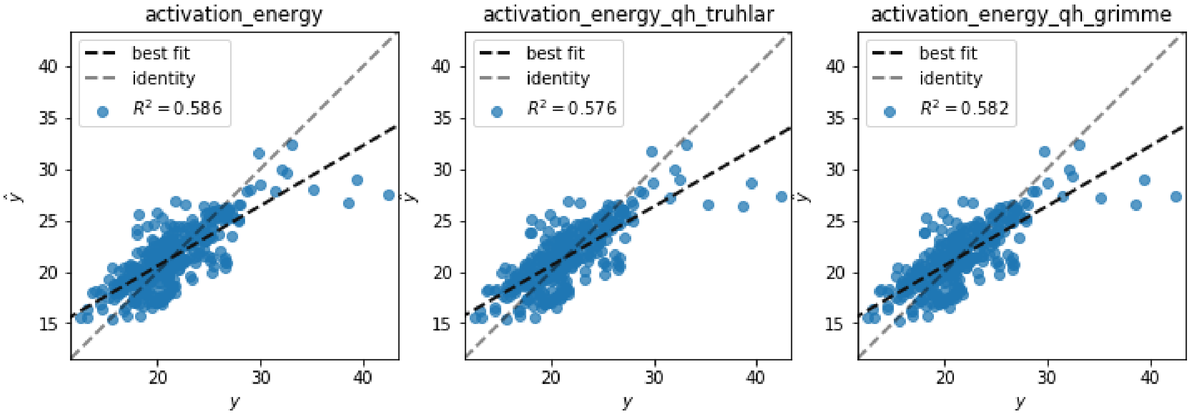

Figure S5. Correlation between DFT-computed activation free energy and experimental activation free energies with cluster-continuum solvation of (a) nucleophile and TS, (b) only nucleophile and (c) no molecules. y: $\Delta G^{\ddagger}_{Exp}$ (kcal/mol), $\hat{y}$: $\Delta G^{\ddagger}_{DFT}$ (kcal/mol)

We used the bias-corrected bootstrap cross-validation (BBC-CV) method for model selection, with the goal to avoid doing nested cross-validation. BBC-CV is a more economical alternative to nested cross-validation that allows unbiased comparison between different families of algorithms.[59] It can also estimate the selection bias for choosing the best estimator based on the cross-validation scores. Empirical studies have found this estimate to give slightly worse scores as compared to the final evaluation on an external test set.[60] For methods where hyperparameter tuning was done via grid search, all resulting models were grouped within a family (*e.g.*, random forest, SVR etc.), and BBC-CV was used to estimate the bias due to overfitting on the cross-validation procedure within this family.[61] This bias was then subtracted from the score of the best model in the family before the scores of each family were

compared. Hyperparameter tuning was done using grid search, with the *GridSearchCV* object in *scikit-learn*, picking the model with the best performance within each family of models. All models were evaluated on the same 10 cross-validation folds, which were generated at the beginning of the BBC-CV procedure. To create interaction features and polynomial features for linear models, we used the *PolynomialFeatures* object in *scikit-learn* with order to second order. Standardization was applied to numeric features, and excluded categorical or count features such as fingerprints.

The full results showed that the $GPR_{M3/2}$ was the strongest contender on the MAE score and we therefore chose it as our final model to be evaluated on the external test set (Table S5). A selection of methods , used in Figure 6 of the manuscript, are given on the same scale in Figure S6. All models from Figure 6 in the manuscript on the same scale..

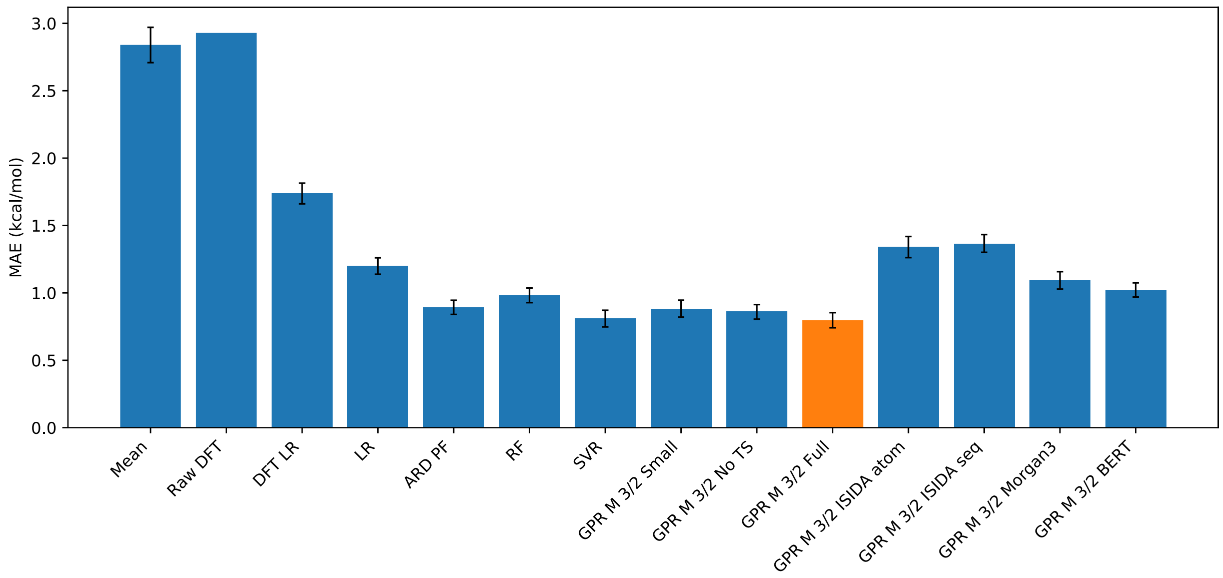

Figure S6. All models from Figure 6 in the manuscript on the same scale.

Table S5. Results for machine learning models. Uncertainty intervals are given as one standard error of the mean. Abbreviations explained in Section 3.1.1. Best method for each score marked in bold.

**Method**	**Feature set**	**R^2 (kcal/mol)**	**MAE (kcal/mol)**	**RMSE (kcal/mol)**
Baseline				
DFT	–	0.09	2.93	3.73
DFT linear fit	–	$0.63^{+0.03}_{-0.03}$	$1.74^{+0.07}_{-0.08}$	$2.36^{+0.15}_{-0.16}$
Mean	–	–	$2.84^{+0.13}_{-0.13}$	$3.90^{+0.24}_{-0.25}$
Median	–	–	$2.81^{+0.13}_{-0.13}$	$3.93^{+0.24}_{-0.24}$
Machine learning models				
Linear regression	Full	$0.79^{+0.03}_{-0.03}$	$1.20^{+0.06}_{-0.06}$	$1.77^{+0.13}_{-0.14}$
KNN	Full	$0.74^{+0.04}_{-0.03}$	$1.37^{+0.07}_{-0.07}$	$1.95^{+0.08}_{-0.12}$
Bayesian ridge	Full	$0.77^{+0.03}_{-0.03}$	$1.24^{+0.06}_{-0.07}$	$1.83^{+0.14}_{-0.14}$
	PF2	$0.69^{+0.09}_{-0.06}$	$1.23^{+0.08}_{-0.10}$	$2.16^{+0.24}_{-0.26}$
PLS	Full	$0.79^{+0.03}_{-0.02}$	$1.25^{+0.06}_{-0.06}$	$1.79^{+0.13}_{-0.13}$
	PF2	$0.84^{+0.03}_{-0.02}$	$0.98^{+0.05}_{-0.05}$	$1.54^{+0.12}_{-0.12}$
ARD	Full	$0.79^{+0.03}_{-0.02}$	$1.21^{+0.06}_{-0.06}$	$1.76^{+0.11}_{-0.11}$
	PF2	$0.85^{+0.03}_{-0.02}$	$0.89^{+0.05}_{-0.05}$	$1.46^{+0.14}_{-0.13}$
Random forest	Full	$0.84^{+0.02}_{-0.02}$	$0.98^{+0.05}_{-0.06}$	$1.53^{+0.11}_{-0.10}$
Gradient boosting	Full	$0.84^{+0.03}_{-0.02}$	$1.00^{+0.06}_{-0.06}$	$1.57^{+0.12}_{-0.13}$
SVR	Full	$0.85^{+0.02}_{-0.02}$	$0.81^{+0.06}_{-0.06}$	$1.48^{+0.15}_{-0.15}$
GPR M 3/2	Full	$\mathbf{0.87^{\pm 0.02}_{-0.02}}$	$\mathbf{0.80^{+0.06}_{-0.06}}$	$1.41^{+0.14}_{-0.14}$
GPR M 5/2	Full	$\mathbf{0.87^{+0.03}_{-0.02}}$	$0.81^{+0.05}_{-0.06}$	$1.40^{+0.14}_{-0.13}$

GPR RQ	Full	$\mathbf{0.87^{+0.03}_{-0.02}}$	$0.83^{+0.05}_{-0.06}$	$1.41^{+0.13}_{-0.13}$
GPR RBF	Full	$\mathbf{0.87^{+0.02}_{-0.02}}$	$0.83^{+0.05}_{-0.05}$	$1.40^{+0.14}_{-0.14}$
Reduced feature sets				
GPR M 3/2	Small	$0.84^{+0.03}_{-0.02}$	$0.88^{+0.06}_{-0.06}$	$1.53^{+0.15}_{-0.14}$
	No TS	$0.86^{+0.02}_{-0.02}$	$0.86^{+0.05}_{-0.05}$	$1.44^{+0.14}_{-0.12}$
	Only TS	$0.83^{+0.03}_{-0.03}$	$1.03^{+0.06}_{-0.06}$	$1.68^{+0.14}_{-0.13}$
	Surface	$0.80^{+0.03}_{-0.03}$	$1.10^{+0.07}_{-0.07}$	$1.76^{+0.15}_{-0.14}$
	Traditional	$0.81^{+0.04}_{-0.03}$	$1.00^{+0.06}_{-0.06}$	$1.68^{+0.16}_{-0.15}$
Structural information				
GPR M 3/2	OHE	$0.62^{+0.04}_{-0.04}$	$1.58^{+0.09}_{-0.09}$	$2.39^{+0.23}_{-0.23}$
	Morgan1	$0.68^{+0.03}_{-0.03}$	$1.55^{+0.08}_{-0.08}$	$2.20^{+0.12}_{-0.13}$
	Morgan2	$0.74^{+0.03}_{-0.03}$	$1.34^{+0.07}_{-0.07}$	$1.94^{+0.17}_{-0.15}$
	Morgan3	$0.80^{+0.03}_{-0.03}$	$1.09^{+0.06}_{-0.06}$	$1.72^{+0.24}_{-0.19}$
	Morgan4	$0.79^{+0.03}_{-0.03}$	$1.13^{+0.07}_{-0.07}$	$1.76^{+0.22}_{-0.18}$
	Morgan5	$0.79^{+0.03}_{-0.03}$	$1.15^{+0.07}_{-0.07}$	$1.81^{+0.23}_{-0.19}$
	Morgan6	$0.78^{+0.03}_{-0.03}$	$1.19^{+0.07}_{-0.07}$	$1.81^{+0.23}_{-0.18}$
	ISIDA atom 4	$0.71^{+0.04}_{-0.03}$	$1.34^{+0.08}_{-0.08}$	$2.09^{+0.13}_{-0.12}$
	ISIDA atom 8	$0.52^{+0.06}_{-0.06}$	$1.64^{+0.09}_{-0.10}$	$2.66^{+0.29}_{-0.29}$
	ISIDA seq 6	$0.74^{+0.04}_{-0.03}$	$1.37^{+0.07}_{-0.06}$	$1.96^{+0.10}_{-0.10}$
	ISIDA seq 8	$0.69^{+0.04}_{-0.03}$	$1.39^{+0.08}_{-0.08}$	$2.15^{+0.14}_{-0.15}$
	BERT pre-trained	$0.85^{+0.02}_{-0.02}$	$1.03^{+0.05}_{-0.05}$	$1.51^{+0.10}_{-0.10}$
	BERT fine-tuned	$0.77^{+0.03}_{-0.03}$	$1.29^{+0.06}_{-0.06}$	$1.82^{+0.10}_{-0.09}$
Structural + physical organic				
GPR M 3/2	Full + Morgan 3	$0.86^{+0.03}_{-0.02}$	$0.87^{+0.05}_{-0.06}$	$1.43^{+0.12}_{-0.13}$
GPR M 3/2	Full + BERT pre-trained	$\mathbf{0.87^{+0.03}_{-0.02}}$	$0.86^{+0.05}_{-0.05}$	$\mathbf{1.34^{+0.11}_{-0.12}}$

3.1.1 Models tested

Support vector regression (SVR).[62] We used the RBF kernel and features were standardized. A grid search was carried out to optimize the values of the kernel coefficient *γ* and the regularization parameter *C*, both with the same range of values (0.001000, 0.004642, 0.02154, 0.1000, 0.4642, 2.154, 10.00, 46.42, 215.4, 1000).

Random forest regression (RF).[63] The number of trees (10, 20, 100, 200) and maximum tree depth (5, 10, 15, 20, None) were optimized.

Gradient boosting regression (GB).[64] We used 1000 boosting stages and early stopping with a threshold of 10 iterations without improvement. A grid search optimized the learning rate (0.001, 0.01, 0.1) and maximum depth of each individual tree estimator (1, 2, 3, 4, 5).

Gaussian Process Regression (GPR).[65] We scaled both the features and the target using the *TransformedTargetRegressor* in scikit-learn. We tried the radial basis function (RBF), rational quadratic (RQ) and Matern 3/2 and 5/2 (M 3/2, M 5/2) kernels. The final composite kernel was created by multiplying with a constant kernel and adding a white kernel to model the noise. An introduction to Gaussian Process Regression in the context of chemistry has been given by Segall and co-workers.[66]

Bayesian ridge regression (BRR).[67] This Bayesian version of ridge regression tunes the regularization parameter automatically from the data.

Automatic Relevance Determination (ARD).[68] ARD produces a more sparse solution than BRR as is suitable when a large number of features are used.

Partial Least Squares (PLS).[69] A linear method that can handle multicollinearity and a large number of features compared to samples. We used a grid search between 1 and 15 to select the number of components.

K-nearest neighbours regression (KNN).[70] Serves as a non-parametric baseline method.

Dummy regression models. We used the mean and the median of the training set for future prediction as reasonable baseline models. These models were implemented with *DummyRegressor* in *scikit-learn*.

3.2 Dataset visualization

The full feature set $\boldsymbol{X}_{full}$ was decomposed with Principal components analysis (PCA)[71] and the two components with highest explained variance were used for the visualization. For the PLS + UMAP plots, we first used PLS for supervised dimensionality reduction. The number of components were chosen based on the R^2 score, using the one-standard error rule to select the model with fewest dimension with a score within one standard error of the best-scoring model overall. This approach led to a model with 5 components. The $\boldsymbol{X}_{full}$ feature space was transformed into this five-dimensional latent space of the PLS model, and further reduced to two dimension with the Uniform Manifold Approximation and Projection (UMAP) method,[72] using the *UMAP* python package,[73] with standard settings, except the keyword *min_dist=0.3*, and *n_neighbors=10*. UMAP is a non-linear dimensionality reduction technique that is frequently used for visualization of high-dimensional data. A reaction map (Figure S7) was generate with the *tmap* package (1.0.4)[74,75] and visualized with *faerun* (0.3.10).[76,77] The Jupyter notebook "reaction_map.ipynb" in the folder "reaction_map" gives the details of how this map was generated. An interactive version of the reaction map is given in the file "index.html".

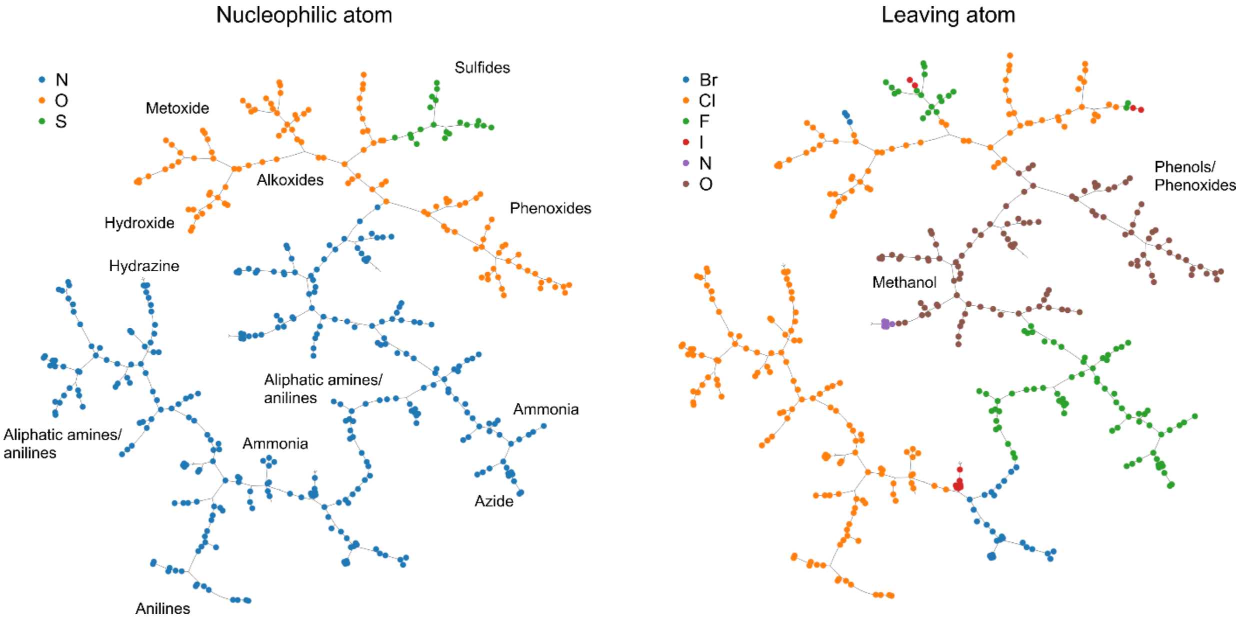

Figure S7. Reaction map generated with $\boldsymbol{X}_{BERT}$, *tmap* and *faerun*. Annotations of nucleophile and leaving group types has been done manually.

3.3 Feature importances

Before assessing the feature importances, we checked the variance inflation factors (Figure S8–Figure S10) and Pearson correlation matrices (Figure S11–Figure S13) to assess the extent of collinearity among the features in $\boldsymbol{X}_{full}$ and $\boldsymbol{X}_{noTS}$ and $\boldsymbol{X}_{small}$. Variance influence factors are a measure of collinearity for each feature, with a value of 1 indicating no collinearity and values above 5–10 considered problematic.[58] It is clear that $\boldsymbol{X}_{full}$ and $\boldsymbol{X}_{noTS}$ have large problems with collinearity, especially in comparison with $\boldsymbol{X}_{small}$. We therefore decided to cluster the features with *SciPy* (1.2.1)[78] based on the Spearman rank correlation,[79]

using the Ward linkage and the distance criterion (following a recipe from the scikit-learn documentation). The threshold for clustering was selected manually to reduce the number of highly correlated features while retaining a clear chemical interpretation of each cluster (Figure S14–Figure S16). We computed the permutation importances version of feature importances with the $GPR_{M3/2}$ model together with the *permutation_importances* function from scikit-learn. We used 10 repeats, and the MAE score and the stability of the feature ranking was assessed by running 10 bootstrap samples with *BootstrapOutOfBag* in *mlxtend.*[80]

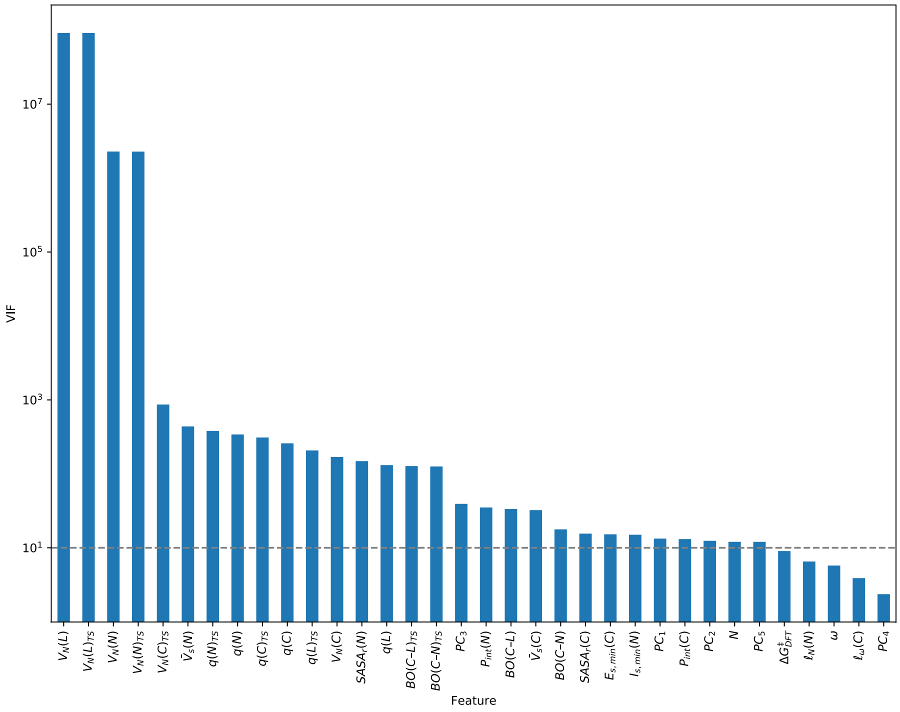

Figure S8. VIFs for $\boldsymbol{X}_{full}$. A value above 10 is indicative of high collinearity with other features. Note log scale.

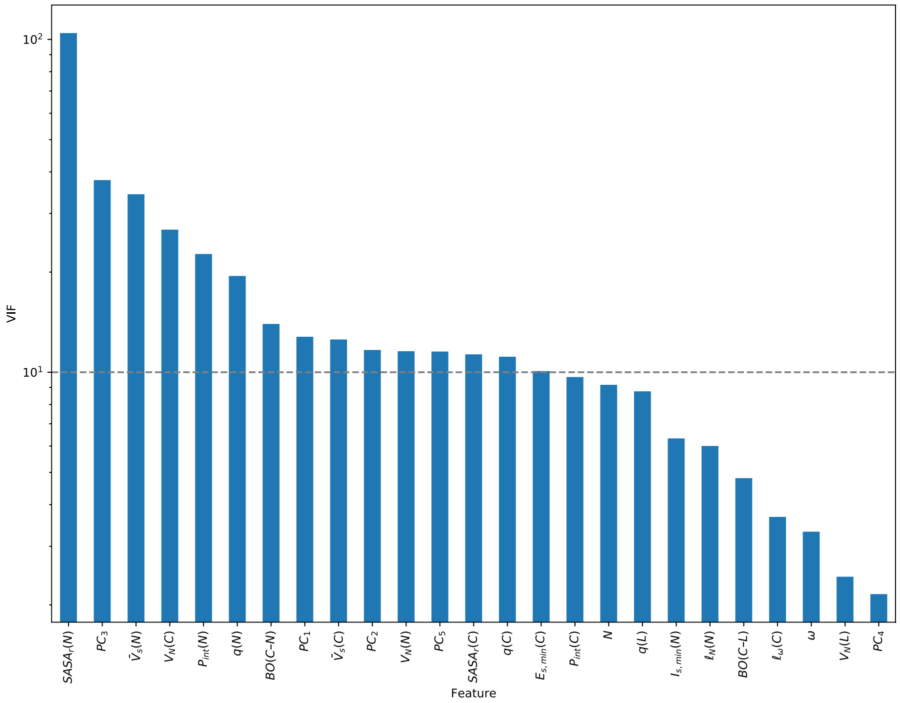

Figure S9. VIFs for $\boldsymbol{X}_{noTS}$. A value above 10 is indicative of high collinearity with other features. Note log scale.

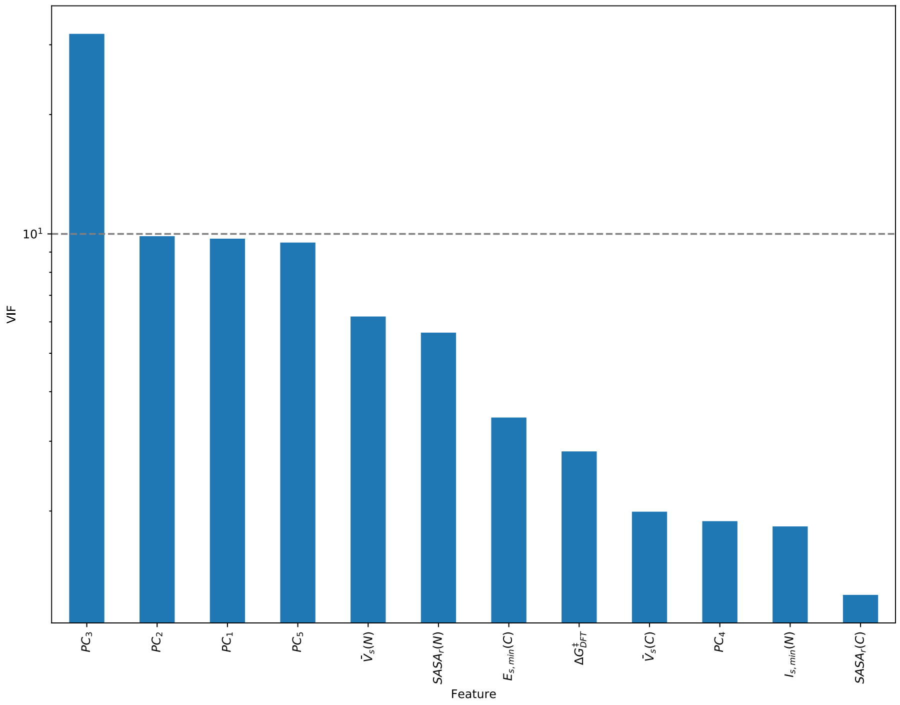

Figure S10. VIFs for $\boldsymbol{X}_{small}$. A value above 10 is indicative of high collinearity with other features. Note log scale.

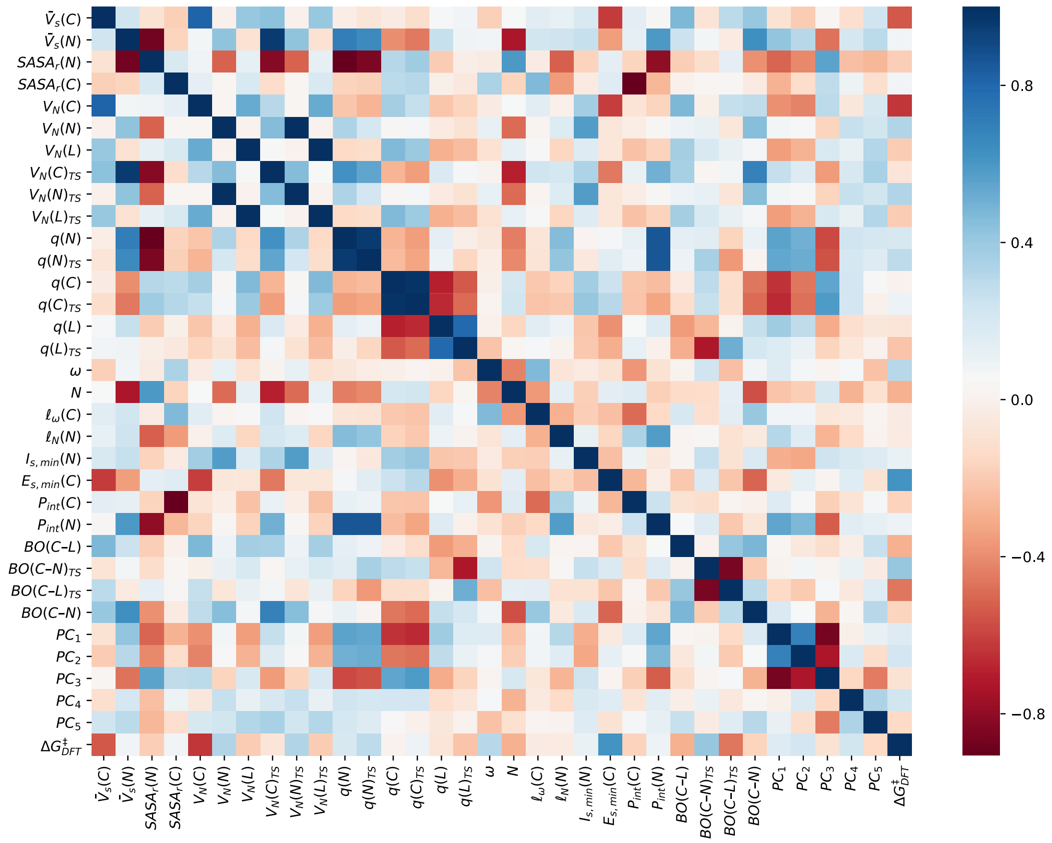

Figure S11. Pearson correlation matrix for $\boldsymbol{X}_{full}$.

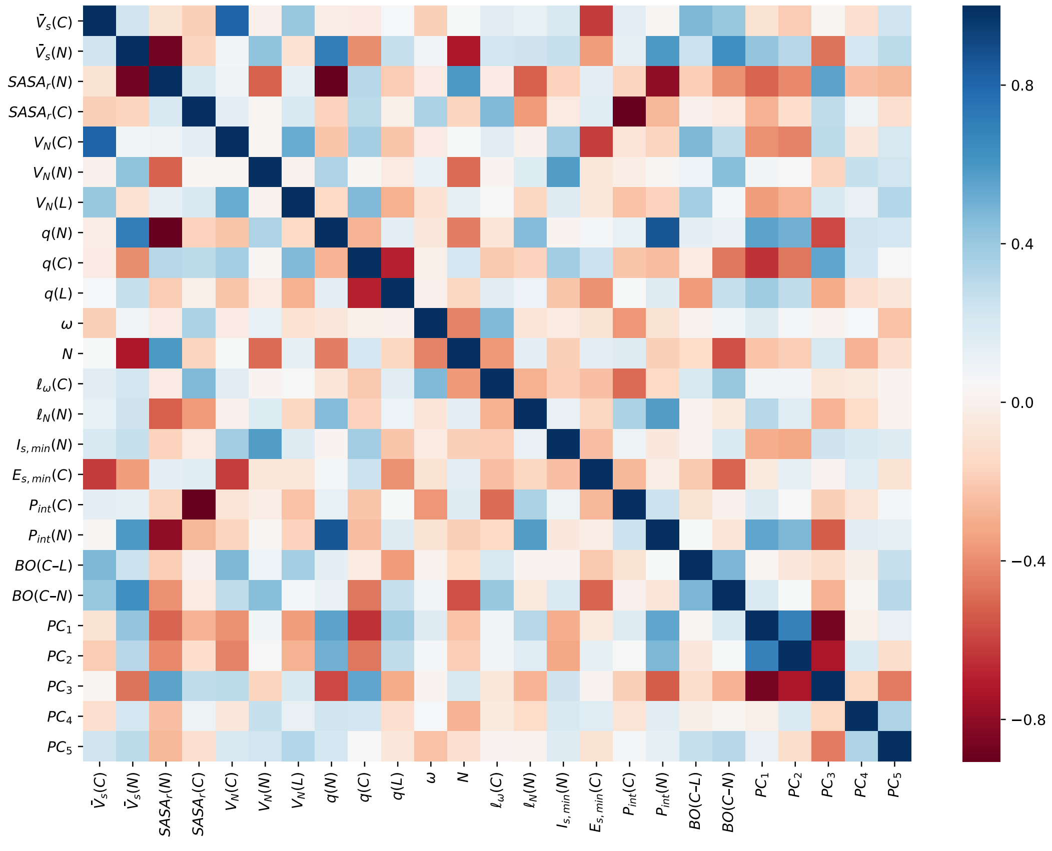

Figure S12. Pearson correlation matrix for $\boldsymbol{X}_{noTS}$.

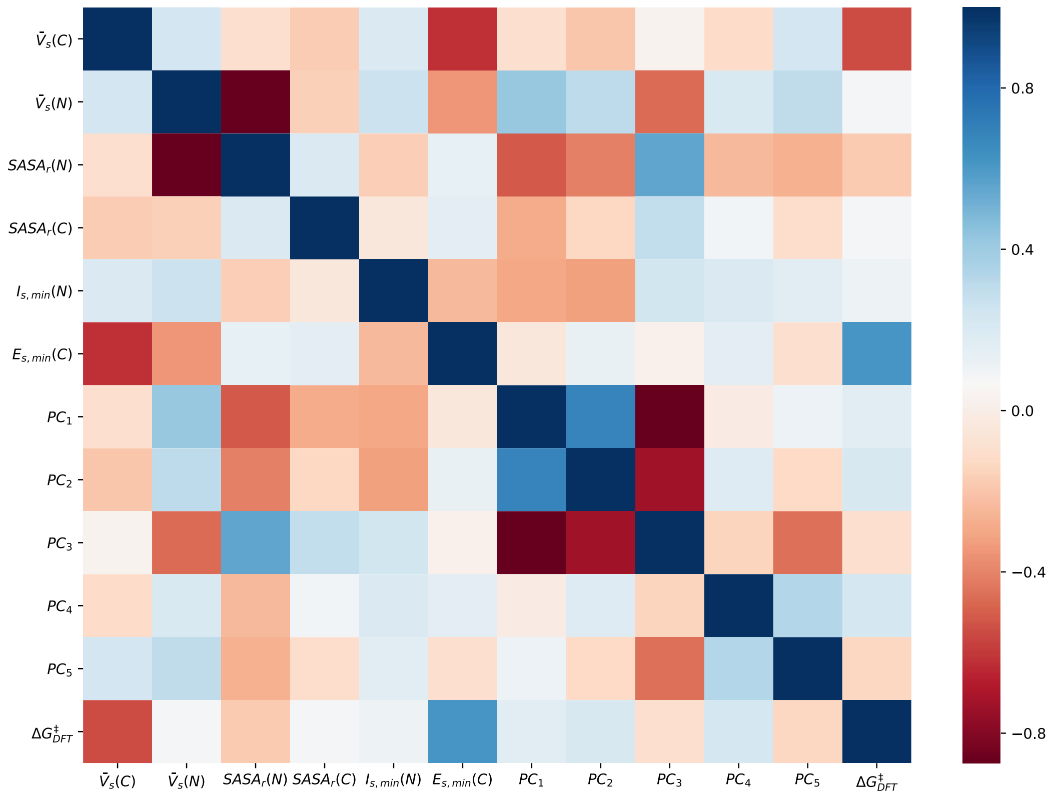

Figure S13. Pearson correlation matrix for $\boldsymbol{X}_{small}$.

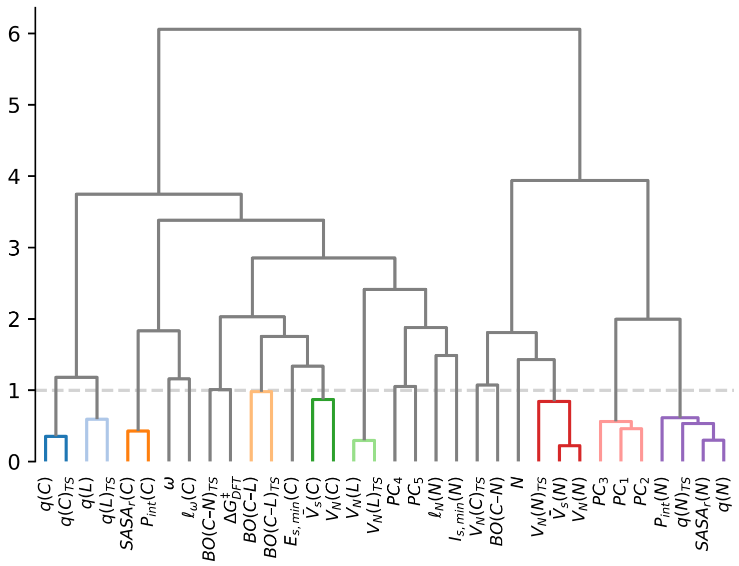

Figure S14. Feature clustering dendrogram for $\boldsymbol{X}_{full}$.

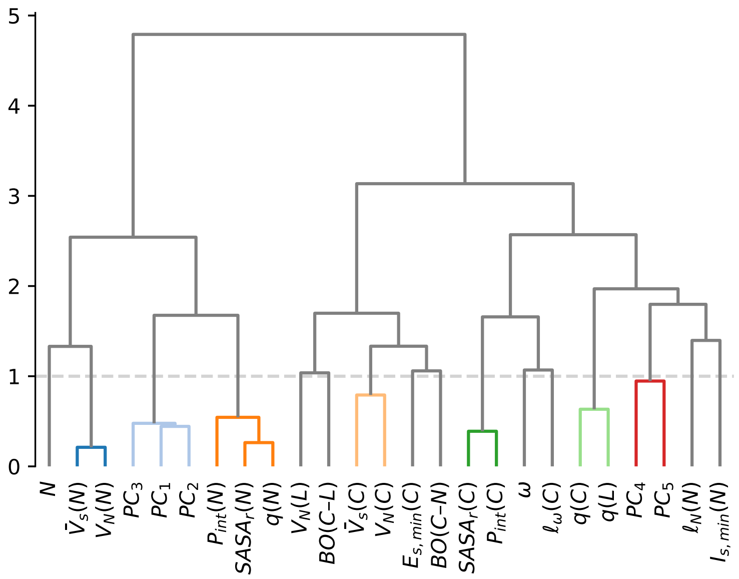

Figure S15. Feature clustering dendrogram for $\boldsymbol{X}_{noTS}$.

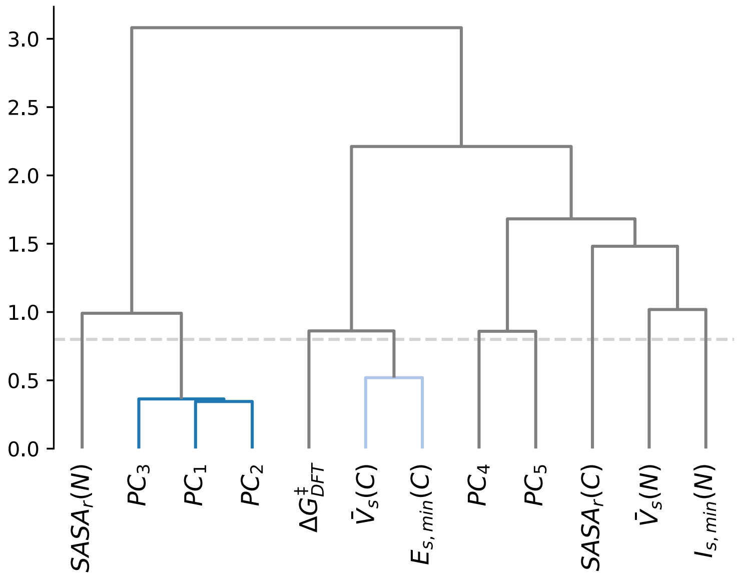

Figure S16. Feature clustering dendrogram for $\boldsymbol{X}_{small}$.

3.4 Learning curves

Learning curves were calculated with the *learning_curve* function in *scikit-learn* with splits of 10%, 20%, 30%, 40%, 50%, 60%, 70%, 80%, 90% and 100% of the data using 10-fold cross-validation. To assess how much data is needed to accurately predict the DFT activation energy, we used $\boldsymbol{X}_{noTS}$ as the feature set.

3.5 Analysis of reactions with large errors

Reactions with absolute residuals larger than 2.0 kcal/mol for the training and test sets with the $GPR_{M3/2}$ model are given in Table S6 and Table S7, respectively.

Table S6. Reactions with error larger than 2.0 kcal/mol in the training set.

**Reaction**	**Residual error (kcal/mol)**

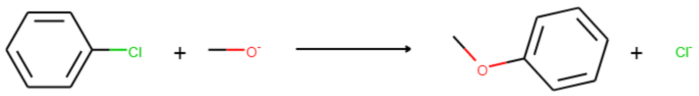	−5.00
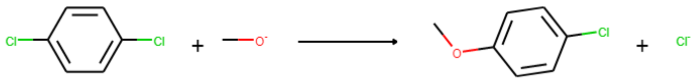	−2.90
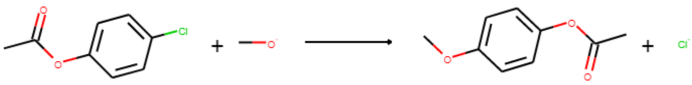	5.10
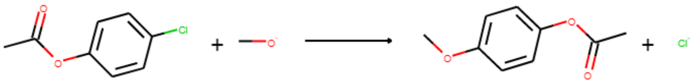	4.23
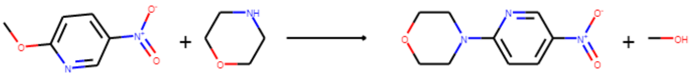	−2.25
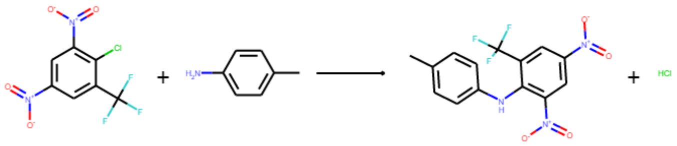	−3.50
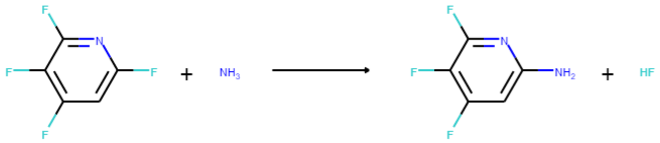	5.52
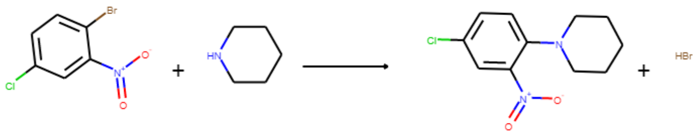	2.27
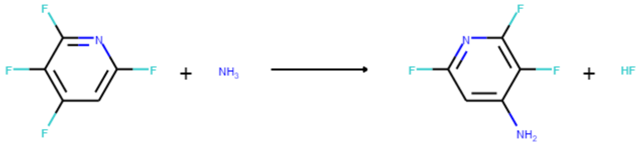	4.41
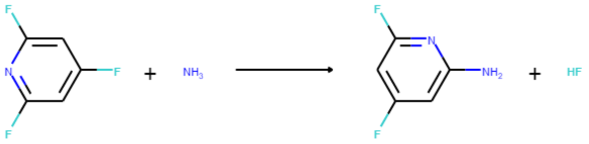	−2.41
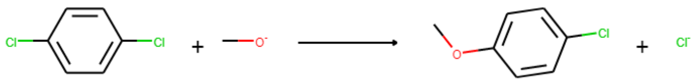	−2.58

Table S7. Reactions with error larger than 2.0 kcal/mol in the test set.

Reaction	Residual error (kcal/mol)

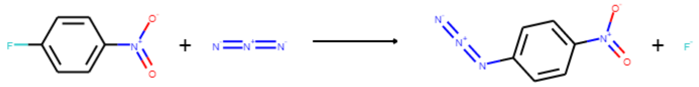	−2.11
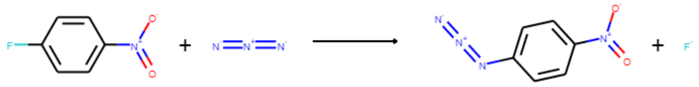	−2.22
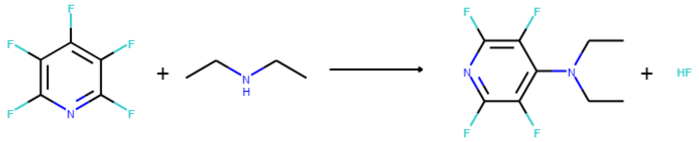	−2.50
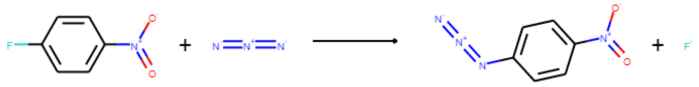	2.06
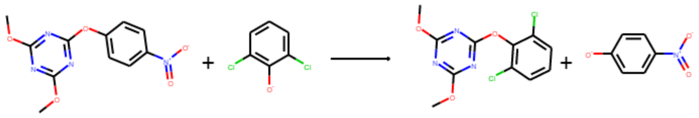	−2.48
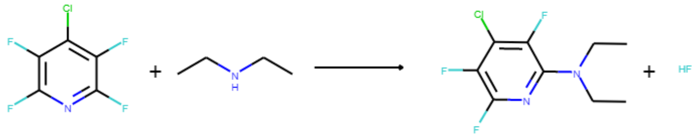	−2.39
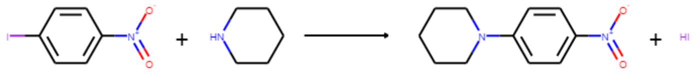	−2.52
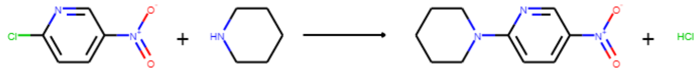	2.27

3.6 Dependence on number of heavy atoms

We checked the dependence of the model error on the number of heavy atoms to see if more complex molecules would display larger error, but could see no such trend (Figure S17).

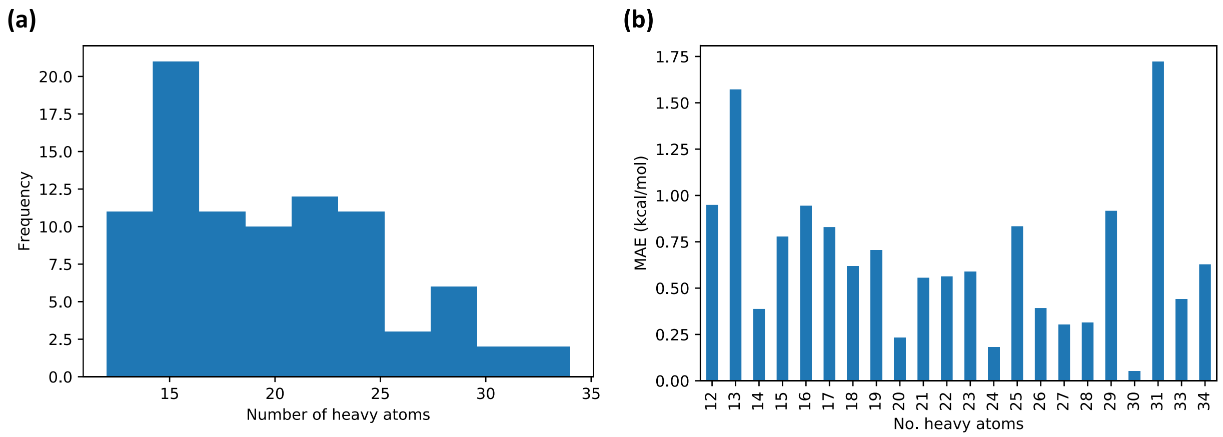

Figure S17. (a) Distribution of number of heavy atoms in the data set. (b) Mean absolute error for test set depending on the number of heavy atoms.

3.7 Y-randomization

We further tested for overfitting by y-randomization, in which the link between the feature vectors in $\boldsymbol{X}$ and the experimental activation free energies in the response vector $\boldsymbol{y}$ is broken by randomizing the $\boldsymbol{y}$ vector (Table S8).[81] The R^2 for $GPR_{M1.5}$ is lowered from 0.86 to −0.05, indicating complete lack of learning when $\boldsymbol{y}$ is randomized. This is strong evidence that the models are not just learning noise but are finding a real signal in the feature data. We performed the y-randomization using the *permutation_test_score* function in scikit-learn with 10 permutations and 10-fold cross-validation for each permutation, as recommended in the literature.[82]

Table S8. Results of y-randomization with $GPR_{M1.5}$ on $\boldsymbol{X}_{full}$ for the training set.

	R^2	MAE (kcal/mol)	RMSE (kcal/mol)
True score	0.86	0.79	1.35
y-randomized score	−0.05	2.87	3.88

4 Regio- and chemoselectivity validation

We used a reaction dataset from the patent literature, collected by Landrum and co-workers.[83] The dataset ("dataSetB.csv") comprised 50,000 reactions that were first classified with the *NameRxn* (3.1.9)[84] tool and then filtered according to reaction classes that include S_NAr reactions according the S_NAr "concept" provided by NextMove (Table S9). Reactions with transition metals were excluded as they would likely go via the Buchwald-Hartwig reaction. The dataset was further filtered manually to remove reactions which apparently did not go via the S_NAr mechanism (*e.g.*, S_N2 reactions). Reactions including boronic acids or phosphine ligands (presumed to occur via Buchwald-Hartwig) were also removed. Furthermore, some reactions also included the product among the starting material and were removed.

Table S9. Reaction types including in the S_NAr concept used to filter out S_NAr reactions from the patent data.

Code	Reaction name
1.3.1	Bromo Buchwald-Hartwig amination
1.3.2	Chloro Buchwald-Hartwig amination
1.3.3	Iodo Buchwald-Hartwig amination
1.3.4	Triflyloxy Buchwald-Hartwig amination
1.3.5	Chan-Lam arylamine coupling
1.3.6	Bromo N-arylation
1.3.7	Chloro N-arylation
1.3.8	Fluoro N-arylation
1.3.9	Iodo N-arylation
1.3.10	Triflyloxy N-arylation
1.3.11	Chichibabin amination
1.3.12	Mesyl N-arylation
1.3.13	Mesyloxy N-arylation
1.3.14	Tosyloxy N-arylation
1.7.11	SNAr ether synthesis
9.7.96	Sandmeyer bromination
9.7.97	Sandmeyer chlorination
9.7.98	Sandmeyer fluorination
9.7.99	Sandmeyer iodination
1.1.2	Menshutkin reaction
1.8.5	Thioether synthesis
9.7.106	Fluoro to azido
9.7.166	Formyl to cyano
9.7.39	Chloro to amino
9.7.44	Chloro to hydroxy
9.7.64	Fluoro to amino

The pruned set of reactions comprised 4353 S_NAr reactions, of which 1209 had alternative reactive sites with halogens on the reactive ring, leading to questions relating to regio- and chemoselectivity (Figure S18). These reactions were sorted according to the molecular weight of the product, and all possible reactions relating to regio- and chemoselectivity of the reactive ring were generated for the 100 reactions with lowest product molecular weight. Sorting on molecular weight was done to allow a quickly calculated validation test.

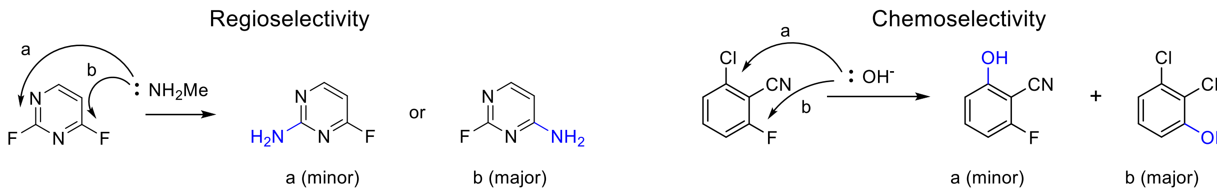

Figure S18. Two examples of reactions with regio- and chemoselectivity questions.

The protonation state of the nucleophile was not included in the patent data, and needed to be set. We used the following set of rules:

1. –OH or –SH nucleophile: Deprotonate
2. Aromatic –NH nucleophile part of diazole or triazole ring:
    a. If strong base present ($pK_a > 10$): Deprotonate
    b. Else: The reactive tautomer is where the nucleophilic atom doesn't have an H
3. Aromatic –NH nucleophile not part of diazole or triazole ring: Deprotonate
4. Non-aromatic -NH:
    a. If strong base present: Deprotonate
    b. Else: Neutral –NH acts as nucleophile.

For a negatively charged nucleophile, the leaving group was also considered to be negatively charged. As reaction solvent and temperature was not available in the dataset, we used a set of standard reaction conditions: acetonitrile for neutral reactions and methanol for ionic reactions and a reaction temperature of 298 K. This is expected to introduce some noise in the predictions. Solvents are sometimes included in the reaction SMILES in the dataset, but it is not always clear what is the reaction solvent and what was used for, *e.g.*, work-up.

For analysis of the results, we constructed the reaction feature matrix $\boldsymbol{X}_{full}$ for the validation reactions and used the previously trained $GPR_{M3/2}$ model for prediction. The isomer corresponding to the reaction with the lowest activation energy was considered as the predicted major product. For comparison, we also used the DFT activation energies.

The validation work is documented in the notebooks listed in Table S10. The validation database is given in the folder "validation_2020_07_04".

Table S10. Jupyter notebooks used for validation work under the folder "validation_dataset".

Notebook file	Description
"validation_data.ipynb"	Generation of the 100 reactions.
"prediction.ipynb"	Prediction with ML method and DFT *vs.* experiment.

5 Experimental dataset

5.1 Description of the modelling data

An extensive literature search was performed to identify intermolecular nucleophilic aromatic substitution reactions with associated second-order kinetic data with a focus on reactions where the

initial nucleophilic attack is rate-limiting. The reaction dataset was extracted from 37 references spanning the years 1950–2019. Kinetic data is not curated by the standard reaction databases, and thus manual extraction and curation was required. In total 518 data points were extracted (the full dataset), and 446 where used in the model building (the modelling dataset). The reason for this smaller number is that 48 of the data points were found after the modelling was finished, 15 had missing data which prevented them from being used, 6 failed in the transition state calculations, two were carried out in a solvent (tetramethylsilane) for which we did not have solvent features, and one was removed due to a mismatch in the reaction SMILES during the modelling process. The whole data set (518 points) covers 88 different nucleophiles, 121 different substrates and 41 different solvents. The experimental temperature range is 273–468 K and log *k*, the base-10 logarithm of the second-order rate constant, spans −15.9 to −3.59. Rate constants were converted to activation energies at the temperature in question using the Eyring equation

$$\Delta G^{\ddagger} = -RT \ln \frac{kh}{k_b T} \qquad \text{Equation S11}$$

where *R* is the gas constant, *T* is the reaction temperature, *k* is the second-order rate constant, *h* is the Planck constant, k_b is the Boltzmann constant and using a standard state of 1 M. Of the reactions reported, 284 have one set of reaction conditions and 70 reactions have more than one set of reaction conditions reported. The partial dataset (446 reactions) used for modelling is described in the main article. A record of this database is given in the files “SNAR_reaction_dataset_SI.csv” and “SNAR_reaction_dataset_SI.xlsx”. Each dataset record includes the following information: canonicalized SMILES of the reaction, reactants and products, second-order rate constants, activation free energy, temperature, solvent, literature source information and flag on its use in the model building process. A full listing of the column names and a corresponding description is given in Table S11.

Table S11. Definitions of columns in full reaction dataset.

Column	Description
Entry	Unique identification number of reaction
Exp_Rate_Constant k1 (M-1s-1)	Experimental second-order rate constant reported in $M^{-1}s^{-1}$
Substrate SMILES	Canonicalized SMILES of substrate
Nucleophile SMILES	Canonicalized SMILES of nucleophile
Product SMILES	Canonicalized SMILES of product
Reaction SMILES	Canonicalized SMILES of reaction
Temp (K)	Reaction temperature in Kelvin
Activation Free Energy (kcalmol-1)	Activation Free energy in $kcalmol^{-1}$ calculated using the experimental rate constant and the Eyring equation
Reference	Journal reference for experimental data
Year	Year of reference
Title	Title of reference
Authors	Authors of reference
DOI	Reference digital object identifier
Book_Page	Page from referenced book where experimental data is extracted from
Table	Table in reference the experimental data is extracted from
Nucleophile	Name of nucleophile
Leaving group	Name of leaving group
Solvent	Solvent for reaction reported.
logk	Base-10 logarithm of second-order rate-constant.
Entry IDs of duplicates	Entry identifiers for duplicate reactions without considerations for reaction conditions.<br>Only four examples with identical reaction conditions: 223 and 468, 27 and 109, 140 and 490, 220 and 469.
Model Flag	“modelled”: Used in the machine learning model. 443 unique reactions with 3 additional replicates.

"not modelled": Reactions that were extracted and added to the data set after model building was completed.
"failed": Reactions that failed the *predict-S_NAr* workflow due to TS finding problems (not finding or finding wrong TS) leading to no barrier, very low barrier, or very high barrier.
"removed": Reactions that were removed for other reasons.
"missing data": Reactions that lack activation energies, temperature or solvent.

5.2 Distribution of activation free energies

The distribution of activation free energies in the modelling dataset is given in Figure S19.

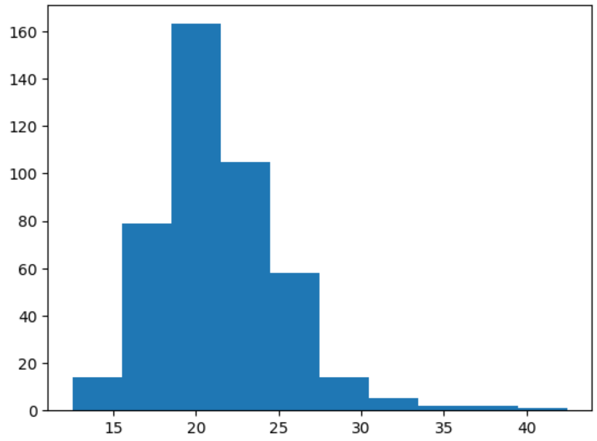

Figure S19. Distribution of activation free energies in the modelling dataset.

5.3 Most common substrates and nucleophiles

The most common substrates and nucleophiles in the modelling dataset are given in Figure S20 and Figure S21.

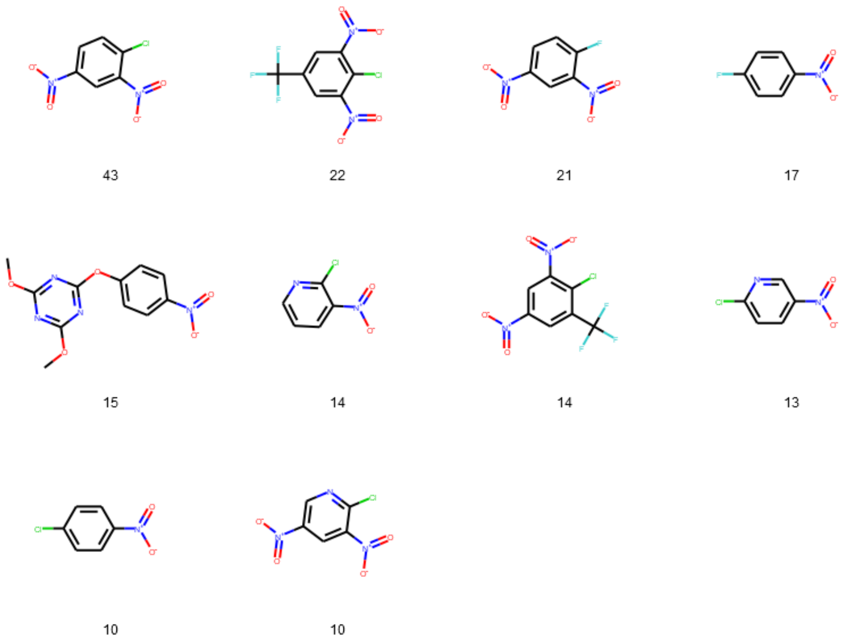

Figure S20. Most common substrates in the dataset with frequency indicated.

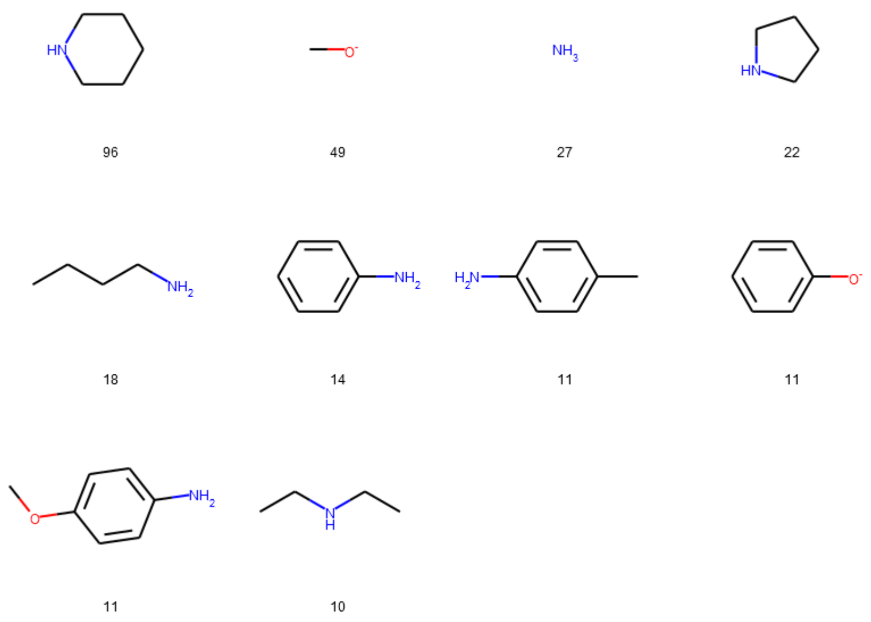

Figure S21. Most common nucleophiles with frequency indicated.

5.4 Replicated reactions

The reactions replicated in the full dataset are given in Table S12, of which the one carried out in TMS is not included in the modelling dataset as solvent features are lacking for the TMS solvent.

Table S12. Replicated reactions in the full dataset.

Reaction	Solvent	Temperature (K)	*ΔG*‡ (kcal/mol)
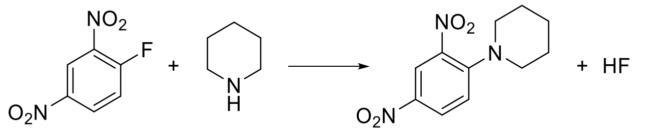	Acetonitrile	298	13.91, 14.46
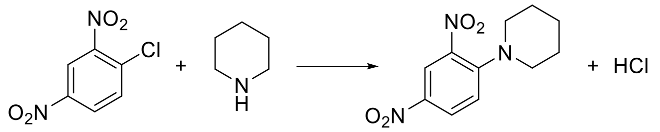	Ethanol	298	19.80, 19.90
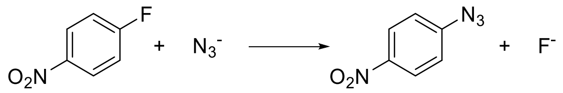	HMPT	298	17.29, 18.92
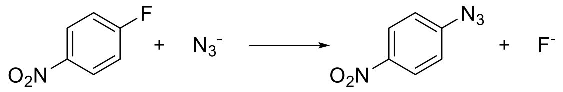	TMS	298	21.18[a], 21.10[a]

[a] Not included in the modelling dataset as we don't have solvent features for TMS.

5.5 Conditions

Conditions are here defined in terms of both solvent and temperature. Figure S22 and Table S13 give the number of reactions with more than one condition in the modelled dataset (446 reactions).

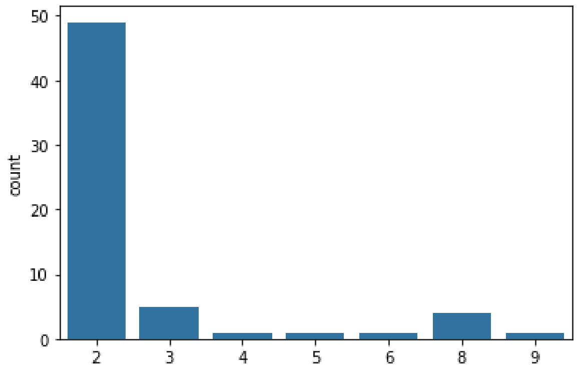

Figure S22. Number of reactions with more than one condition in the modelling dataset.

Table S13. Number of reactions with more than one condition in the modelling dataset.

**No. conditions**	**2**	**3**	**4**	**5**	**6**	**8**	**9**
Counts	49	5	1	1	1	4	1

5.6 Reaction temperatures

The distribution of reactions at different reaction temperatures for the modelling dataset are given in Figure S23 and Table S14. Correlation analysis confirmed that exclusion of reaction temperature was warranted (*R*: 0.64, *ρ*: 0.54 *τ*: 0.43) as it could possibly artificially inflate the performance of the model (Figure S24). Reactions with higher barriers are often run at higher temperatures to be measured in reasonable time.

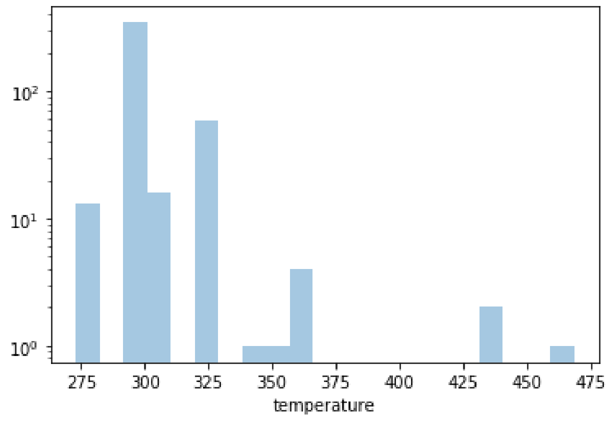

Figure S23. Distribution of reaction with temperature in the modelling dataset. Note log scale.

Table S14. Reactions with a certain temperature in the modelling dataset.

**Temperature**	**298**	**323**	**293**	**303**	**273**	**363**	**432.6**	**468.4**	**432.5**	**353**	**344**
Counts	288	59	58	16	13	4	1	1	1	1	1

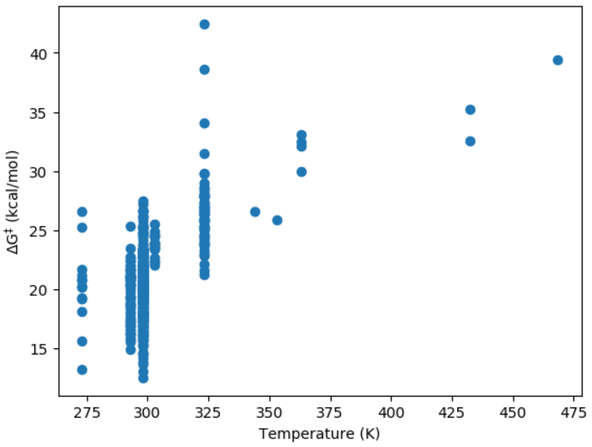

Figure S24. Experimental activation energy as a function of reaction temperature.

5.7 Solvent distribution

The distribution of reaction solvents depending on nucleophile charge and atom type are given in Figure S25. The solvents are given as their SMILES strings. While reactions with neutral nitrogen nucleophiles are carried out in a variety of different solvents, anionic oxygen nucleophiles are only used in water or methanol.

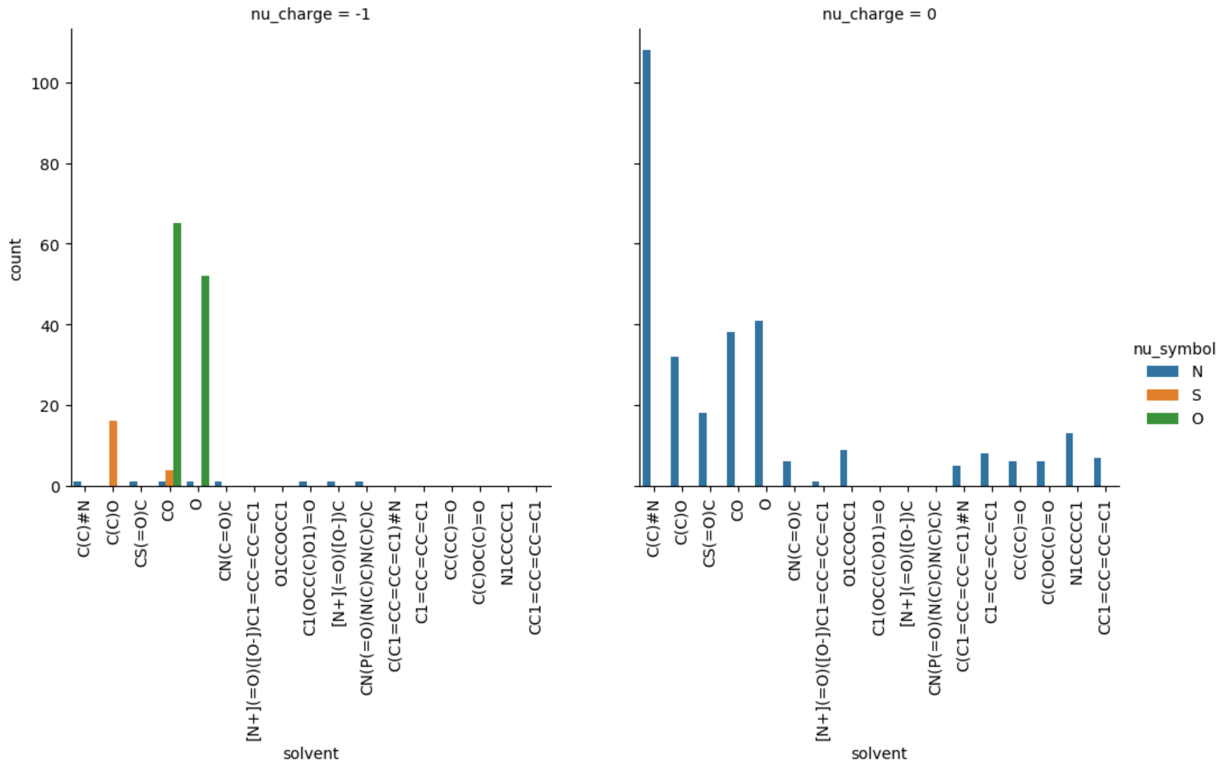

Figure S25. Distribution of reaction solvent depending on nucleophile charge and atom type.

6 Workflow database

The database holding the calculations from the predict-S_NAr workflow is a Python shelve database which operates as a dictionary with keys and values. Each key is a unique identifier, holding a dictionary of the calculation results which are described in Table S15. The database is housed in the folder "machine_learning/2019-12-15" under the name "db". It can be loaded with the following Python code:

```
import shelve
d = shelve.open(“db”)
```

The workflow dataset includes a number of duplicate reactions that need to be removed before modelling (see the notebooks referenced in Section 2).

Table S15. Keys for the workflow dataset dictionary.

Key	Description of values	Datatype
'solvent'	Solvent SMILES string	String
'clustering_energies'	Energy correction from explicit solvent model (a.u.)	See table below
'agent'	Flag for agent (acid/base)	Bool/None
'temperature'	Temperature (K)	Float
'n_cpus'	Number of CPUs	Integer
'run_time'	Run time (seconds)	Float
'end_time'	End time (YYYY-MM-DD HH:MM:SS)	String
'flat_PES'	Flag for flat PES where intermediate is found but not elimination TS	Bool
'intramolecular'	Flag for intramolecular reaction	Bool
'concerted'	Flag for concerted reaction	Bool
'descriptors'	Descriptors	See table below.
'reactive_atoms'	Reactive atoms	Dictionary, keys: string, values: integer
'symbols'	Chemical symbols	Dictionary, keys: string, values: list of strings
'geometries'	Geometries (Å)	Dictionary, keys: string, values: list of floats
'entropy_corr_qh_truhlar'	Entropy terms with Truhlar quasi-harmonic correction (a.u.)	Dictionary, keys: string, values: float
'entropy_corr_qh_grimme'	Entropy terms with Grimme quasi-harmonic correction (a.u.)	Dictionary, keys: string, values: float
'entropy_corr'	Entropy terms (a.u.)	Dictionary, keys: string, values: float
'enthalpy_corr'	Enthalpy terms (a.u.)	Dictionary, keys: string, values: float
'free_energies_qh_truhlar'	Free energies with Truhlar quasi-harmonic correction (a.u.)	Dictionary, keys: string, values: float
'free_energies_qh_grimme'	Free energies with Grimme quasi-harmonic correction (a.u.)	Dictionary, keys: string, values: float
'free_energies'	Free energies (a.u.)	Dictionary, keys: string, values: float
'enthalpies'	Enthalpies (a.u.)	Dictionary, keys: string, values: float
'electronic_energies'	Electronic energies (a.u.)	Dictionary, keys: string, values: float
'inchi_key'	InChIKey	Dictionary, keys: string, values: string
'inchi'	InChI	Dictionary, keys: string, values: string
'smiles'	SMILES	String

The keys used in the sub-dictionaries are described in Table S16.

Table S16. Keys for sub-dictionaries in the workflow dataset.

Key	Description
“substrate”	Substrate
“nucleophile”	Nucleophile
“product”	Product
“leaving_group”	Leaving group or leaving atom
“ts”	Transition state
“agent”	Agent. Not implemented.
“reaction”	Related to the reaction, e.g., reaction SMILES
“reaction_orig”	Related to the reaction as originally input, e.g., reaction SMILES
“solvent”	Solvent

The keys for the descriptor sub-dictionary are given in Table S17.

Table S17. Keys for the descriptor sub-dictionary.

Key	Description	Value
'epn'	Electrostatic potential at nuclei (V_N, a.u.)	Dictionary, keys: integer, values: float
'hirshfeld'	Hirshfeld atomic charge	Dictionary, keys: integer, values: float
'hirshfeld_plus'	Hirshfeld atomic charge for cation	Dictionary, keys: integer, values: float
'hirshfeld_minus'	Hirshfeld atomic charge for anion	Dictionary, keys: integer, values: float
'ddec6_charge'	DDEC6 charge (*q*)	Dictionary, keys: integer, values: float
'ddec6_bo'	DDEC6 bond order (*BO*)	Dictionary, keys: tuple of integers, values: float
'v_av'	Atom surface average of the electrostatic potential ($\overline{V}_s$, kcal/mol)	Dictionary, keys: integer, values: float
'vs_max'	Atom surface maximum of the electrostatic potential (kcal/mol).	Dictionary, keys: integer, values: float
'vs_min'	Atom surface minimum of the electrostatic potential (kcal/mol).	Dictionary, keys: integer, values: float
'es_min_b3lyp'	Atomic surface minimum of the local electron attachment with the B3LYP functional ($E_{s,}$min, eV).	Dictionary, keys: integer, values: float
'es_min_blyp'	Atomic surface minimum of the local electron attachment with the BLYP functional (eV).	Dictionary, keys: integer, values: float
'is_min'	Atomic surface minimum of the the average local ionization energy ($I_{s,min}$, eV).	Dictionary, keys: integer, values: float
'sasa'	Solvent accessible surface area (Å^2)	Dictionary, keys: integer, values: float
'sasa_ratio'	Ratio of available solvent accessible surface area ($SASA_r$)	Dictionary, keys: integer, values: float
'ip'	Ionization potential (eV)	Float
'ea'	Ionization potential (eV)	Float
'atom_p_int'	Atomic dispersion potentials (kcal$^{0.5}$ mol$^{-0.5}$)	Dictionary, keys: integer, values: float
'atom_p_int_area'	Atomic dispersion potentials multiplied by atomic area (kcal$^{0.5}$ mol$^{-0.5}$ Å^2)	Dictionary, keys: integer, values: float
'p_int'	Average dispersion potential of entire molecule (kcal$^{0.5}$ mol$^{-0.5}$)	Float
'p_int_area'	Average dispersion potential of entire molecule multiplied by its area (kcal$^{0.5}$ mol$^{-0.5}$ Å^2)	Float

The subdictionary “clustering_energies” contains the solvent corrections from explicit solvation and are explained in Table S18. It uses the keys 'xtb' and 'dft'.

Table S18. Keys for the clustering energies sub-dictionary

Key	Description
'clustering_energy'	Solvation model correction (a.u.)
'clustering_energy_qh_grimme'	Solvation model correction with Grimme correction (a.u.)
'clustering_energy_qh_truhlar'	Solvation model correction with Truhlar correction (a.u.)

7 Analysis package

A Python package, *analyze_snar*, was written for extraction of data from the workflow database as well as machine learning routines and statistical functions. It is included with the manuscript and can be installed with “`pip install .`”.

8 References

1 https://www.daylight.com/dayhtml/doc/theory/theory.smiles.html.

2 C. Reichardt, *Solvents and Solvent Effects in Organic Chemistry*, Wiley-VCH, Weinheim, 3rd edn., 2003.

3 D. M. Lowe, P. T. Corbett, P. Murray-Rust and R. C. Glen, *J. Chem. Inf. Model.*, 2011, **51**, 739–753.

4 https://github.com/dan2097/opsin.

5 L. J. Diorazio, D. R. J. Hose and N. K. Adlington, *Org. Process Res. Dev.*, 2016, **20**, 760–773.
6 RDKit: Open-source cheminformatics, http://www.rdkit.org.
7 J.-P. Ebejer, G. M. Morris and C. M. Deane, *J. Chem. Inf. Model.*, 2012, **52**, 1146–1158.
8 S. Riniker and G. A. Landrum, *J. Chem. Inf. Model.*, 2015, **55**, 2562–2574.
9 T. A. Halgren, *J. Comput. Chem.*, 1996, **17**, 490–519.
10 P. Tosco, N. Stiefl and G. Landrum, *J. Cheminformatics*, 2014, **6**, 37.
11 A. K. Rappe, C. J. Casewit, K. S. Colwell, W. A. Goddard and W. M. Skiff, *J. Am. Chem. Soc.*, 1992, **114**, 10024–10035.
12 M. J. Frisch, G. W. Trucks, H. B. Schlegel, G. E. Scuseria, M. A. Robb, J. R. Cheeseman, G. Scalmani, V. Barone, G. A. Petersson, H. Nakatsuji, X. Li, M. Caricato, A. V. Marenich, J. Bloino, B. G. Janesko, R. Gomperts, B. Mennucci, H. P. Hratchian, J. V. Ortiz, A. F. Izmaylov, J. L. Sonnenberg, Williams, F. Ding, F. Lipparini, F. Egidi, J. Goings, B. Peng, A. Petrone, T. Henderson, D. Ranasinghe, V. G. Zakrzewski, J. Gao, N. Rega, G. Zheng, W. Liang, M. Hada, M. Ehara, K. Toyota, R. Fukuda, J. Hasegawa, M. Ishida, T. Nakajima, Y. Honda, O. Kitao, H. Nakai, T. Vreven, K. Throssell, J. A. Montgomery Jr., J. E. Peralta, F. Ogliaro, M. J. Bearpark, J. J. Heyd, E. N. Brothers, K. N. Kudin, V. N. Staroverov, T. A. Keith, R. Kobayashi, J. Normand, K. Raghavachari, A. P. Rendell, J. C. Burant, S. S. Iyengar, J. Tomasi, M. Cossi, J. M. Millam, M. Klene, C. Adamo, R. Cammi, J. W. Ochterski, R. L. Martin, K. Morokuma, O. Farkas, J. B. Foresman and D. J. Fox, *Gaussian 16 Rev. C.01*, Wallingford, CT, 2016.
13 J.-D. Chai and M. Head-Gordon, *Phys. Chem. Chem. Phys.*, 2008, **10**, 6615–6620.
14 R. Ditchfield, W. J. Hehre and J. A. Pople, *J. Chem. Phys.*, 1971, **54**, 724–728.
15 T. Clark, J. Chandrasekhar, G. W. Spitznagel and P. V. R. Schleyer, *J. Comput. Chem.*, 1983, **4**, 294–301.
16 N. Chéron, D. Jacquemin and P. Fleurat-Lessard, *Phys. Chem. Chem. Phys.*, 2012, **14**, 7170–7175.
17 S. Grimme, *Chem. - Eur. J.*, 2012, **18**, 9955–9964.
18 G. Luchini, J. V. Alegre-Requena, I. Funes-Ardoiz and R. S. Paton, *F1000Research*, 2020, **9**, 291.
19 https://github.com/bobbypaton/GoodVibes.
20 R. Krishnan, J. S. Binkley, R. Seeger and J. A. Pople, *J. Chem. Phys.*, 1980, **72**, 650–654.
21 A. V. Marenich, C. J. Cramer and D. G. Truhlar, *J. Phys. Chem. B*, 2009, **113**, 6378–6396.
22 C. Bannwarth, S. Ehlert and S. Grimme, *J. Chem. Theory Comput.*, 2019, **15**, 1652–1671.
23 https://github.com/grimme-lab/xtb.
24 J. R. Pliego and J. M. Riveros, *J. Phys. Chem. A*, 2001, **105**, 7241–7247.
25 J. R. Pliego Jr and J. M. Riveros, *Wiley Interdiscip. Rev. Comput. Mol. Sci.*, 2019, e1440.
26 https://github.com/grimme-lab/crest.
27 R. E. Plata and D. A. Singleton, *J. Am. Chem. Soc.*, 2015, **137**, 3811–3826.
28 F. Terrier, *Modern Nucleophilic Aromatic Substitution*, Wiley-VCH, Weinheim, Germany, 2013.
29 F. Weigend and R. Ahlrichs, *Phys. Chem. Chem. Phys.*, 2005, **7**, 3297–3305.
30 D. Rappoport and F. Furche, *J. Chem. Phys.*, 2010, **133**, 134105.
31
32 A. Shrake and J. A. Rupley, *J. Mol. Biol.*, 1973, **79**, 351–371.
33 W. M. Haynes and W. M. Haynes, *CRC Handbook of Chemistry and Physics*, 95th edn., 2014.
34 M. Rahm, R. Hoffmann and N. W. Ashcroft, *Chem. - Eur. J.*, 2016, **22**, 14625–14632.
35 S. Grimme, J. Antony, S. Ehrlich and H. Krieg, *J. Chem. Phys.*, 2010, **132**, 154104.
36 T. Brinck, P. Carlqvist and J. H. Stenlid, *J. Phys. Chem. A*, 2016, **120**, 10023–10032.
37 P. Sjoberg, J. S. Murray, T. Brinck and P. Politzer, *Can. J. Chem.*, 1990, **68**, 1440–1443.

38 T. Brinck, *HS95: Molecular surface property program*, .
39 T. A. Manz and N. Gabaldon Limas, *Chargemol program for performing DDEC analysis*, 2017.
40 https://sourceforge.net/projects/ddec/.
41 R. Contreras, J. Andres, V. S. Safont, P. Campodonico and J. G. Santos, *J. Phys. Chem. A*, 2003, **107**, 5588–5593.
42 R. G. Parr, L. v. Szentpály and S. Liu, *J. Am. Chem. Soc.*, 1999, **121**, 1922–1924.
43 J. Oller, P. Pérez, P. W. Ayers and E. Vöhringer-Martinez, *Int. J. Quantum Chem.*, 2018, **118**, e25706.
44 F. L. Hirshfeld, *Theor. Chim. Acta*, 1977, **44**, 129–138.
45 *BIOVIA Pipeline Pilot*, Dassault Systèmes, San Diego, 2018.
46 R. I. Nugmanov, R. N. Mukhametgaleev, T. Akhmetshin, T. R. Gimadiev, V. A. Afonina, T. I. Madzhidov and A. Varnek, *J. Chem. Inf. Model.*, 2019, **59**, 2516–2521.
47 https://github.com/cimm-kzn/CGRtools.
48 https://github.com/cimm-kzn/CIMtools.
49 Philippe Schwaller, Daniel Probst, Alain C. Vaucher, Vishnu H Nair, Teodoro Laino and Jean-Louis Reymond, *ChemRxiv*, , DOI:10.26434/chemrxiv.9897365.v2.
50 https://github.com/rxn4chemistry/rxnfp/.
51 A. Hjorth Larsen, J. Jørgen Mortensen, J. Blomqvist, I. E. Castelli, R. Christensen, M. Dułak, J. Friis, M. N. Groves, B. Hammer, C. Hargus, E. D. Hermes, P. C. Jennings, P. Bjerre Jensen, J. Kermode, J. R. Kitchin, E. Leonhard Kolsbjerg, J. Kubal, K. Kaasbjerg, S. Lysgaard, J. Bergmann Maronsson, T. Maxson, T. Olsen, L. Pastewka, A. Peterson, C. Rostgaard, J. Schiøtz, O. Schütt, M. Strange, K. S. Thygesen, T. Vegge, L. Vilhelmsen, M. Walter, Z. Zeng and K. W. Jacobsen, *J. Phys. Condens. Matter*, 2017, **29**, 273002.
52 N. M. O'boyle, A. L. Tenderholt and K. M. Langner, *J. Comput. Chem.*, 2008, **29**, 839–845.
53 F. Pedregosa, G. Varoquaux, A. Gramfort, V. Michel, B. Thirion, O. Grisel, M. Blondel, P. Prettenhofer, R. Weiss, V. Dubourg, J. Vanderplas, A. Passos, D. Cournapeau, M. Brucher, M. Perrot and E. Duchesnay, *J. Mach. Learn. Res.*, 2011, **12**, 2825–2830.
54 W. McKinney, in *Proceedings of the 9th Python in Science Conference*, eds. S. van der Walt and J. Millman, 2010, pp. 56–61.
55 J. D. Hunter, *Comput. Sci. Eng.*, 2007, **9**, 90–95.
56 https://github.com/mwaskom/seaborn.
57 K. Pearson, *Proc. R. Soc. Lond.*, 1895, **58**, 240–242.
58 G. James, D. Witten, T. Hastie and R. Tibshirani, *An introduction to statistical learning: with applications in R*, Springer, New York, 2013.
59 I. Tsamardinos, E. Greasidou and G. Borboudakis, *Mach. Learn.*, 2018, **107**, 1895–1922.
60 G. Borboudakis, T. Stergiannakos, M. Frysali, E. Klontzas, I. Tsamardinos and G. E. Froudakis, *Npj Comput. Mater.*, 2017, **3**, 40.
61 G. C. Cawley and N. L. Talbot, *J. Mach. Learn. Res.*, 2010, **11**, 2079–2107.
62 B. E. Boser, I. M. Guyon and V. N. Vapnik, in *Proceedings of the Fifth Annual Workshop on Computational Learning Theory*, Association for Computing Machinery, New York, NY, USA, 1992, pp. 144–152.
63 L. Breiman, *Mach. Learn.*, 2001, **45**, 5–32.
64 J. H. Friedman, *Ann Stat.*, 2001, **29**, 1189–1232.
65 C. E. Rasmussen and C. K. I. Williams, *Gaussian processes for machine learning*, MIT Press, Cambridge, Mass, 2006.
66 O. Obrezanova, G. Csányi, J. M. R. Gola and M. D. Segall, *J. Chem. Inf. Model.*, 2007, **47**, 1847–1857.

67 R. M. Neal, *Bayesian learning for neural networks*, Springer, New York, 1996.
68 D. P. Wipf and S. S. Nagarajan, in *Advances in Neural Information Processing Systems 20*, eds. J. C. Platt, D. Koller, Y. Singer and S. T. Roweis, Curran Associates, Inc., 2008, pp. 1625–1632.
69 S. Wold, M. Sjöström and L. Eriksson, *Chemom. Intell. Lab. Syst.*, 2001, **58**, 109–130.
70 N. S. Altman, *Am. Stat.*, 1992, **46**, 175–185.
71 K. Pearson, *Lond. Edinb. Dublin Philos. Mag. J. Sci.*, 1901, **2**, 559–572.
72 L. McInnes, J. Healy and J. Melville, *arXiv:1802.03426*.
73 L. McInnes, J. Healy, N. Saul and L. Großberger, *J. Open Source Softw.*, 2018, **3**, 861.
74 D. Probst and J.-L. Reymond, *J. Cheminformatics*, 2020, **12**, 12.
75 https://github.com/reymond-group/tmap.
76 D. Probst and J.-L. Reymond, *Bioinformatics*, 2018, **34**, 1433–1435.
77 https://github.com/reymond-group/faerun.
78 SciPy 1.0 Contributors, P. Virtanen, R. Gommers, T. E. Oliphant, M. Haberland, T. Reddy, D. Cournapeau, E. Burovski, P. Peterson, W. Weckesser, J. Bright, S. J. van der Walt, M. Brett, J. Wilson, K. J. Millman, N. Mayorov, A. R. J. Nelson, E. Jones, R. Kern, E. Larson, C. J. Carey, İ. Polat, Y. Feng, E. W. Moore, J. VanderPlas, D. Laxalde, J. Perktold, R. Cimrman, I. Henriksen, E. A. Quintero, C. R. Harris, A. M. Archibald, A. H. Ribeiro, F. Pedregosa and P. van Mulbregt, *Nat. Methods*, 2020, **17**, 261–272.
79 C. Spearman, *Am. J. Psychol.*, 1904, **15**, 72.
80 S. Raschka, *J. Open Source Softw.*, , DOI:10.21105/joss.00638.
81 C. Rücker, G. Rücker and M. Meringer, *J. Chem. Inf. Model.*, 2007, **47**, 2345–2357.
82 M. M. C. Ferreira, in *Encyclopedia of Physical Organic Chemistry*, American Cancer Society, 2017, pp. 1–38.
83 N. Schneider, N. Stiefl and G. A. Landrum, *J. Chem. Inf. Model.*, 2016, **56**, 2336–2346.
84 *NameRxn*, NextMove Software Limited, 2019.
