# Supplementary material for: Machine learning meets mechanistic modelling for accurate prediction of experimental activation energies
Source: Chem Sci. 2020 Nov 5;12(3):1163–75. doi: 10.1039/d0sc04896h (PMC9528810; doi:10.1039/d0sc04896h)
Supplement: SC-012-D0SC04896H-s002 [file SC-012-D0SC04896H-s002.zip › SC-012-D0SC04896H-s153.html]

>


0 / 0
